# Supplementary material for: Effects of Loading Forces, Loading Positions, and Splinting of Two, Three, or Four Ti-Zr (Roxolid®) Mini-Implants Supporting the Mandibular Overdentures on Peri-Implant and Posterior Edentulous Area Strains
Source: J Funct Biomater. 2024 Sep 9;15(9):260. doi: 10.3390/jfb15090260 (PMC11433416; doi:10.3390/jfb15090260)
Supplement: Supplementary file 1 [file jfb-15-00260-s001.zip › jfb-3107962-supplementary.pdf]

Supplementary Table 1. Descriptive statistics (mean values and standard deviations of periimplant microstrains registered when two mini-implants either as single-units or splinted) when they supported mandibular overdentures; N=Number

Strain gauge position	Force	Splinting status	Loading position	Mean	Std. Deviation	N
Right MDI Vestibular Strain gauge	50 N	Not splinted (single-units)	Bilateral	592.28	105.05	15
			Anterior	544.89	47.55	15
			Unilateral (right side)	1454.05	53.43	15
		Splinted	Bilateral	445.36	89.91	15
			Anterior	398.78	44.69	15
			Unilateral (right side)	1298.45	47.77	15
	100 N	Not splinted (single-units)	Bilateral	803.64	56.50	15
			Anterior	865.43	56.74	15
			Unilateral (right side)	1629.88	55.87	15
		Splinted	Bilateral	646.75	53.99	15
			Anterior	712.84	59.50	15
			Unilateral (right side)	1477.61	56.62	15
	150 N	Not splinted (single-units)	Bilateral	1051.32	63.96	15
			Anterior	1543.73	74.42	15
			Unilateral (right side)	1836.64	62.99	15
		Splinted	Bilateral	902.45	74.13	15
			Anterior	1387.06	48.39	15
			Unilateral (right side)	1693.03	54.65	15
	200 N	Not splinted (single-units)	Bilateral	1646.34	103.34	15
			Anterior	1594.37	57.83	15
			Unilateral (right side)	2503.02	59.38	15
		Splinted	Bilateral	1485.36	100.04	15
			Anterior	1439.79	39.39	15
			Unilateral (right side)	2352.02	44.44	15
	250 N	Not splinted (single-units)	Bilateral	1859.26	67.74	15
			Anterior	1911.76	57.33	15
			Unilateral (right side)	2669.55	68.22	15
		Splinted	Bilateral	1713.60	56.25	15
			Anterior	1782.69	57.23	15
			Unilateral (right side)	2531.02	60.59	15
	300 N	Not splinted (single-units)	Bilateral	2114.51	65.11	15
			Unilateral (right side)	2876.48	61.33	15
		Splinted	Bilateral	1962.00	74.22	15
			Unilateral (right side)	2730.94	49.27	15
Left MDI Vestibular Strain gauge	50 N	Not splinted (single-units)	Bilateral	569.83	83.93	15
			Anterior	551.59	82.63	15
			Unilateral (right side)	1039.17	61.23	15
		Splinted	Bilateral	418.55	82.48	15
			Anterior	392.52	64.58	15
			Unilateral (right side)	893.06	62.22	15
	100 N	Not splinted (single-units)	Bilateral	767.61	60.86	15
			Anterior	829.93	53.55	15
			Unilateral (right side)	1294.77	76.91	15
		Splinted	Bilateral	609.78	64.23	15
			Anterior	693.09	50.51	15

			Unilateral (right side)	1125.51	53.52	15
	150 N	Not splinted (single-units)	Bilateral	1071.21	53.40	15
			Anterior	1539.51	86.63	15
			Unilateral (right side)	1444.48	54.57	15
		Splinted	Bilateral	908.81	53.08	15
			Anterior	1390.98	68.70	15
			Unilateral (right side)	1302.74	55.92	15
	200 N	Not splinted (single-units)	Bilateral	1631.64	98.31	15
			Anterior	1595.47	85.74	15
			Unilateral (right side)	2099.77	65.43	15
		Splinted	Bilateral loading	1465.72	81.84	15
			Anterior loading	1454.31	71.59	15
			Unilateral (right side)	1941.31	75.99	15
	250 N	Not splinted (single-units)	Bilateral loading	1828.71	68.44	15
			Anterior loading	1880.93	59.15	15
			Unilateral (right side)	2358.04	82.38	15
		Splinted	Bilateral loading	1666.79	77.01	15
			Anterior loading	1739.22	60.87	15
			Unilateral (right side)	2173.83	60.82	15
	300 N	Not splinted (single-units)	Bilateral loading	2115.30	71.53	15
			Unilateral (right side)	2489.84	67.83	15
		Splinted	Bilateral loading	1956.25	59.83	15
			Unilateral (right side)	2344.11	67.25	15
**Right MDI Oral Strain gauge**	50 N	Not splinted (single-units)	Bilateral	556.04	79.90	15
			Anterior loading	491.79	40.13	15
			Unilateral (right side)	1334.25	73.37	15
		Splinted	Bilateral	404.81	71.30	15
			Anterior loading	336.35	25.32	15
			Unilateral (right side)	1183.03	61.13	15
	100 N	Not splinted (single-units)	Bilateral	668.82	80.65	15
			Anterior loading	679.00	57.27	15
			Unilateral (right side)	1546.38	56.81	15
		Splinted	Bilateral	528.09	84.25	15
			Anterior loading	537.22	50.69	15
			Unilateral (right side)	1388.12	63.21	15
	150 N	Not splinted (single-units)	Bilateral	865.50	68.22	15
			Anterior loading	1503.82	64.34	15
			Unilateral (right side)	1726.63	81.28	15
		Splinted	Bilateral	711.40	57.77	15
			Anterior loading	1329.17	52.16	15
			Unilateral (right side)	1580.99	65.98	15
	200 N	Not splinted (single-units)	Bilateral	1596.88	73.86	15
			Anterior loading	1550.50	47.69	15
			Unilateral (right side))	2390.62	83.24	15
		Splinted	Bilateral	1448.16	80.57	15
			Anterior loading	1380.93	38.06	15
			Unilateral (right side)	2238.62	58.03	15
	250 N	Not splinted (single-units)	Bilateral	1726.33	87.47	15
			Anterior loading	1727.59	56.58	15
			Unilateral (right side)	2597.46	58.29	15
		Splinted	Bilateral	1574.42	79.15	15
			Anterior loading	1594.36	60.01	15
			Unilateral (right side)	2426.26	60.45	15
	300 N	Not splinted (single-units)	Bilateral	1914.59	66.11	15
			Unilateral (right side)	2775.59	87.44	15

		Splinted	Bilateral	1753.05	77.22	15
			Unilateral (right side)	2628.34	75.21	15
**Left MDI Oral Strain gauge**	50 N	Not splinted (single-units)	Bilateral	465.05	20.22	15
			Anterior loading	504.75	64.34	15
			Unilateral (right side)	994.98	57.58	15
		Splinted	Bilateral	322.96	12.50	15
			Anterior loading	353.63	54.31	15
			Unilateral (right side)	851.08	56.94	15
	100 N	Not splinted (single-units)	Bilateral	690.80	64.17	15
			Anterior loading	673.78	55.69	15
			Unilateral (right side)	1267.99	34.62	15
		Splinted	Bilateral	536.78	49.97	15
			Anterior loading	527.30	51.54	15
			Unilateral (right side)	1113.37	35.15	15
	150 N	Not splinted (single-units)	Bilateral	824.68	78.46	15
			Anterior loading	1492.98	86.20	15
			Unilateral (right side)	1332.27	80.56	15
		Splinted	Bilateral	679.14	82.09	15
			Anterior loading	1363.12	66.58	15
			Unilateral (right side)	1179.11	86.48	15
	200 N	Not splinted (single-units)	Bilateral	1508.38	37.90	15
			Anterior loading	1560.30	69.85	15
			Unilateral (right side)	2052.10	52.95	15
		Splinted	Bilateral	1357.43	31.68	15
			Anterior loading	1414.28	70.65	15
			Unilateral (right side)	1907.63	61.74	15
	250 N	Not splinted (single-units)	Bilateral	1747.86	59.22	15
			Anterior loading	1715.33	55.37	15
			Unilateral (right side)	2322.81	45.50	15
		Splinted	Bilateral	1586.59	54.21	15
			Anterior loading	1575.98	49.80	15
			Unilateral (right side)	2156.82	30.73	15
	300 N	Not splinted (single-units)	Bilateral	1868.03	77.42	15
			Unilateral (right side)	2393.43	96.89	15
		Splinted	Bilateral	1727.84	94.18	15
			Unilateral (right side)	2232.31	79.09	15

Supplementary Table 2. Multivariate analysis (tests of between-subjects effects) of periimplant microstrains (dependent variables) registered in the 2-mini-implant models with mini-implants having different splinting status (single-units or splinted) loaded with forces from 50-300 N at different loading positions (splinting status, loading forces and loading positions = factors)

Multivariate analysis: Tests of Between-Subjects Effects

Source	Strain gauge position	Type III Sum of Squares	df	Mean Square	F	Sig.	Partial Eta Squared
Corrected Model	Right MDI Vestibular SG	234597483.707[a]	33	7109014.66	1704.66	<0.001**	.992
	Left MDI Vestibular SG	179853824.437[b]	33	5450115.89	1133.26	<0.001**	.987
	Right MDI Oral SG	232186361.470[c]	33	7035950.35	1566.63	<0.001**	.991
	Left MDI Oral SG	180983697.624[d]	33	5484354.47	1410.66	<0.001**	.990
Intercept	Right MDI Vestibular SG	1220540766.33	1	1220540766.33	292671.46	<0.001**	.998
	Left MDI Vestibular SG	1015158420.57	1	1015158420.57	211085.71	<0.001**	.998
	Right MDI Oral SG	1051463122.79	1	1051463122.79	234119.49	<0.001**	.998
	Left MDI Oral SG	878456446.76	1	878456446.76	225952.50	<0.001**	.998
Force	Right MDI Vestibular SG	144388291.91	5	28877658.38	6924.53	<0.001**	.986
	Left MDI Vestibular SG	145473759.72	5	29094751.94	6049.78	<0.001**	.985
	Right MDI Oral SG	137594722.13	5	27518944.43	6127.39	<0.001**	.985
	Left MDI Oral SG	136789921.54	5	27357984.31	7036.89	<0.001**	.987
Splinting status	Right MDI Vestibular SG	2786679.02	1	2786679.02	668.21	<0.001**	.584
	Left MDI Vestibular SG	2974287.37	1	2974287.37	618.45	<0.001**	.565
	Right MDI Oral SG	2954138.65	1	2954138.65	657.77	<0.001**	.580
	Left MDI Oral SG	2762987.78	1	2762987.78	710.68	<0.001**	.599
Loading position	Right MDI Vestibular SG	70090870.05	2	35045435.02	8403.49	<0.001**	.972
	Left MDI Vestibular SG	20414838.32	2	10207419.16	2122.47	<0.001**	.899
	Right MDI Oral SG	73062516.13	2	36531258.07	8134.07	<0.001**	.972
	Left MDI Oral SG	27918085.35	2	13959042.68	3590.48	<0.001**	.938
*Force * Splinting status*	Right MDI Vestibular SG	4369.09	5	873.82	0.21	0.958	.002

	Left MDI Vestibular SG	2576.70	5	515.34	0.11	0.991	.001
	Right MDI Oral SG	1797.58	5	359.52	0.08	0.995	.001
	Left MDI Oral SG	2291.78	5	458.36	0.12	0.988	.001
*Force * Loading position*	Right MDI Vestibular SG	4564592.92	9	507176.99	121.62	<0.001**	.697
	Left MDI Vestibular SG	4527764.31	9	503084.92	104.61	<0.001**	.664
	Right MDI Oral SG	6577352.20	9	730816.91	162.72	<0.001**	.755
	Left MDI Oral SG	7789075.82	9	865452.87	222.61	<0.001**	.808
*Splinting status * Loading position*	Right MDI Vestibular SG	524.55	2	262.28	0.06	0.939	.000
	Left MDI Vestibular SG	5324.48	2	2662.24	0.55	0.575	.002
	Right MDI Oral SG	336.46	2	168.23	0.04	0.963	.000
	Left MDI Oral SG	2574.94	2	1287.47	0.33	0.718	.001
*Force * Splinting status * Loading position*	Right MDI Vestibular SG	2244.65	9	249.41	0.06	1.000	.001
	Left MDI Vestibular SG	10897.15	9	1210.79	0.25	0.986	.005
	Right MDI Oral SG	12599.27	9	1399.92	0.31	0.971	.006
	Left MDI Oral SG	5038.32	9	559.81	0.14	0.998	.003
Error	Right MDI Vestibular SG	1985083.88	476	4170.34			
	Left MDI Vestibular SG	2289190.54	476	4809.22			
	Right MDI Oral SG	2137782.02	476	4491.14			
	Left MDI Oral SG	1850589.25	476	3887.79			
Total	Right MDI Vestibular SG	1450579727.16	510				
	Left MDI Vestibular SG	1181086675.06	510				
	Right MDI Oral SG	1280448634.28	510				
	Left MDI Oral SG	1048673325.10	510				
Corrected Total	Right MDI Vestibular SG	236582567.59	509				
	Left MDI Vestibular SG	182143014.98	509				
	Right MDI Oral SG	234324143.49	509				
	Left MDI Oral SG	182834286.88	509				

a. R Squared = .992 (Adjusted R Squared = .991)
b. R Squared = .987 (Adjusted R Squared = .987)
c. R Squared = .991 (Adjusted R Squared = .990)

d. R Squared = .990 (Adjusted R Squared = .989)

Supplementary Table 3. Post hoc Sheffe tests for the variable: Loading Force in the 2-MDI models

MULTIPLE COMPARISONS: FORCE: SCHEFFE POST-HOC							
DEPENDENT VARIABLE	Force		Mean Difference (I-J)	Std. Error	*P*	95% Confidence Interval	
						Lower Bound	Upper Bound
RIGHT MDI VESTIBULAR SG	50 N	100 N	-233.7233*	9.63	<0.01**	-265.89	-201.56
		150 N	-613.4061*	9.63	<0.01**	-645.57	-581.24
		200 N	-1047.8484*	9.63	<0.01**	-1080.01	-1015.68
		250 N	-1289.0109*	9.63	<0.01**	-1321.18	-1256.84
		300 N	-1632.0148*	10.76	<0.01**	-1667.98	-1596.05
	100 N	50 N	233.7233*	9.63	<0.01**	201.56	265.89
		150 N	-379.6828*	9.63	<0.01**	-411.85	-347.52
		200 N	-814.1251*	9.63	<0.01**	-846.29	-781.96
		250 N	-1055.2876*	9.63	<0.01**	-1087.45	-1023.12
		300 N	-1398.2915*	10.76	<0.01**	-1434.25	-1362.33
	150 N	50 N	613.4061*	9.63	<0.01**	581.24	645.57
		100 N	379.6828*	9.63	<0.01**	347.52	411.85
		200 N	-434.4423*	9.63	<0.01**	-466.61	-402.28
		250 N	-675.6048*	9.63	<0.01**	-707.77	-643.44
		300 N	-1018.6086*	10.76	<0.01**	-1054.57	-982.65
	200 N	50 N	1047.8484*	9.63	<0.01**	1015.68	1080.01
		100 N	814.1251*	9.63	<0.01**	781.96	846.29
		150 N	434.4423*	9.63	<0.01**	402.28	466.61
		250 N	-241.1625*	9.63	<0.01**	-273.33	-209.00
		300 N	-584.1663*	10.76	<0.01**	-620.13	-548.20
	250 N	50 N	1289.0109*	9.63	<0.01**	1256.84	1321.18
		100 N	1055.2876*	9.63	<0.01**	1023.12	1087.45
		150 N	675.6048*	9.63	<0.01**	643.44	707.77
		200 N	241.1625*	9.63	<0.01**	209.00	273.33
		300 N	-343.0038*	10.76	<0.01**	-378.97	-307.04
	300 N	50 N	1632.0148*	10.76	<0.01**	1596.05	1667.98
		100 N	1398.2915*	10.76	<0.01**	1362.33	1434.25
		150 N	1018.6086*	10.76	<0.01**	982.65	1054.57

		200 N	584.1663*	10.76	<0.01**	548.20	620.13
		250 N	343.0038*	10.76	<0.01**	307.04	378.97
**LEFT MDI VESTIBULAR SG**	50 N	100 N	-242.6643*	10.34	<0.01**	-277.21	-208.12
		150 N	-632.1706*	10.34	<0.01**	-666.71	-597.63
		200 N	-1053.9193*	10.34	<0.01**	-1088.46	-1019.38
		250 N	-1297.1340*	10.34	<0.01**	-1331.68	-1262.59
		300 N	-1582.2561*	11.56	<0.01**	-1620.88	-1543.64
	100 N	50 N	242.6643*	10.34	<0.01**	208.12	277.21
		150 N	-389.5063*	10.34	<0.01**	-424.05	-354.96
		200 N	-811.2550*	10.34	<0.01**	-845.80	-776.71
		250 N	-1054.4697*	10.34	<0.01**	-1089.01	-1019.93
		300 N	-1339.5918*	11.56	<0.01**	-1378.21	-1300.97
	150 N	50 N	632.1706*	10.34	<0.01**	597.63	666.71
		100 N	389.5063*	10.34	<0.01**	354.96	424.05
		200 N	-421.7487*	10.34	<0.01**	-456.29	-387.21
		250 N	-664.9634*	10.34	<0.01**	-699.51	-630.42
		300 N	-950.0854*	11.56	<0.01**	-988.71	-911.47
	200 N	50 N	1053.9193*	10.34	<0.01**	1019.38	1088.46
		100 N	811.2550*	10.34	<0.01**	776.71	845.80
		150 N	421.7487*	10.34	<0.01**	387.21	456.29
		250 N	-243.2147*	10.34	<0.01**	-277.76	-208.67
		300 N	-528.3368*	11.56	<0.01**	-566.96	-489.72
	250 N	50 N	1297.1340*	10.34	<0.01**	1262.59	1331.68
		100 N	1054.4697*	10.34	<0.01**	1019.93	1089.01
		150 N	664.9634*	10.34	<0.01**	630.42	699.51
		200 N	243.2147*	10.34	<0.01**	208.67	277.76
		300 N	-285.1220*	11.56	<0.01**	-323.74	-246.50
	300 N	50 N	1582.2561*	11.56	<0.01**	1543.64	1620.88
		100 N	1339.5918*	11.56	<0.01**	1300.97	1378.21
		150 N	950.0854*	11.56	<0.01**	911.47	988.71
		200 N	528.3368*	11.56	<0.01**	489.72	566.96
		250 N	285.1220*	11.56	<0.01**	246.50	323.74

**RIGHT MDI ORAL SG**	50 N	100 N	-173.5584*	9.99	<0.01**	-206.94	-140.18
		150 N	-568.5402*	9.99	<0.01**	-601.92	-535.16
		200 N	-1049.9058*	9.99	<0.01**	-1083.29	-1016.52
		250 N	-1223.3603*	9.99	<0.01**	-1256.74	-1189.98
		300 N	-1550.1815*	11.17	<0.01**	-1587.50	-1512.86
	100 N	50 N	173.5584*	9.99	<0.01**	140.18	206.94
		150 N	-394.9818*	9.99	<0.01**	-428.36	-361.60
		200 N	-876.3474*	9.99	<0.01**	-909.73	-842.97
		250 N	-1049.8019*	9.99	<0.01**	-1083.18	-1016.42
		300 N	-1376.6231*	11.17	<0.01**	-1413.94	-1339.30
	150 N	50 N	568.5402*	9.99	<0.01**	535.16	601.92
		100 N	394.9818*	9.99	<0.01**	361.60	428.36
		200 N	-481.3655*	9.99	<0.01**	-514.75	-447.98
		250 N	-654.8201*	9.99	<0.01**	-688.20	-621.44
		300 N	-981.6413*	11.17	<0.01**	-1018.96	-944.32
	200 N	50 N	1049.9058*	9.99	<0.01**	1016.52	1083.29
		100 N	876.3474*	9.99	<0.01**	842.97	909.73
		150 N	481.3655*	9.99	<0.01**	447.98	514.75
		250 N	-173.4545*	9.99	<0.01**	-206.84	-140.07
		300 N	-500.2758*	11.17	<0.01**	-537.60	-462.95
	250 N	50 N	1223.3603*	9.99	<0.01**	1189.98	1256.74
		100 N	1049.8019*	9.99	<0.01**	1016.42	1083.18
		150 N	654.8201*	9.99	<0.01**	621.44	688.20
		200 N	173.4545*	9.99	<0.01**	140.07	206.84
		300 N	-326.8212*	11.17	<0.01**	-364.14	-289.50
	300 N	50 N	1550.1815*	11.17	<0.01**	1512.86	1587.50
		100 N	1376.6231*	11.17	<0.01**	1339.30	1413.94
		150 N	981.6413*	11.17	<0.01**	944.32	1018.96
		200 N	500.2758*	11.17	<0.01**	462.95	537.60
		250 N	326.8212*	11.17	<0.01**	289.50	364.14
**LEFT MDI ORAL SG**	50 N	100 N	-219.5979*	9.29	<0.01**	-250.66	-188.54
		150 N	-563.1428*	9.29	<0.01**	-594.20	-532.09

	200 N	-1051.2798*	9.29	<0.01**	-1082.34	-1020.22
	250 N	-1268.8236*	9.29	<0.01**	-1299.88	-1237.77
	300 N	-1473.3298*	10.39	<0.01**	-1508.05	-1438.61
100 N	50 N	219.5979*	9.29	<0.01**	188.54	250.66
	150 N	-343.5449*	9.29	<0.01**	-374.60	-312.49
	200 N	-831.6819*	9.29	<0.01**	-862.74	-800.62
	250 N	-1049.2258*	9.29	<0.01**	-1080.28	-1018.17
	300 N	-1253.7319*	10.39	<0.01**	-1288.46	-1219.01
150 N	50 N	563.1428*	9.29	<0.01**	532.09	594.20
	100 N	343.5449*	9.29	<0.01**	312.49	374.60
	200 N	-488.1370*	9.29	<0.01**	-519.19	-457.08
	250 N	-705.6808*	9.29	<0.01**	-736.74	-674.62
	300 N	-910.1870*	10.39	<0.01**	-944.91	-875.46
200 N	50 N	1051.2798*	9.29	<0.01**	1020.22	1082.34
	100 N	831.6819*	9.29	<0.01**	800.62	862.74
	150 N	488.1370*	9.29	<0.01**	457.08	519.19
	250 N	-217.5438*	9.29	<0.01**	-248.60	-186.49
	300 N	-422.0500*	10.39	<0.01**	-456.77	-387.33
250 N	50 N	1268.8236*	9.29	<0.01**	1237.77	1299.88
	100 N	1049.2258*	9.29	<0.01**	1018.17	1080.28
	150 N	705.6808*	9.29	<0.01**	674.62	736.74
	200 N	217.5438*	9.29	<0.01**	186.49	248.60
	300 N	-204.5062*	10.39	<0.01**	-239.23	-169.78
300 N	50 N	1473.3298*	10.39	<0.01**	1438.61	1508.05
	100 N	1253.7319*	10.39	<0.01**	1219.01	1288.46
	150 N	910.1870*	10.39	<0.01**	875.46	944.91
	200 N	422.0500*	10.39	<0.01**	387.33	456.77
	250 N	204.5062*	10.39	<0.01**	169.78	239.23

BASED ON OBSERVED MEANS.
THE ERROR TERM IS MEAN SQUARE(ERROR) = 3887.793.
***. THE MEAN DIFFERENCE IS SIGNIFICANT AT THE .01 LEVEL.**
***P*=LEVEL OF SIGNIFICANCE; **=SIGNIFICANT AT P<0.01; NS=NOT SIGNIFICANT**

Supplementary Table 4. Post hoc tests for the independent variable: Loading Position in the 2-MDI models

Multiple Comparisons: Loading Position: Post-hoc Scheffe

Dependent Variable	**Loading Position**		**Mean Difference (I-J)**	**Std. Error**	***P***	**95% Confidence Interval**	
						**Lower Bound**	**Upper Bound**
Right MDI Vestibular SG	Bilateral	Anterior	50.4386*	7.14	<0.01**	32.91	67.97
		Unilateral (right side)	-819.1520*	6.81	<0.01**	-835.87	-802.44
	Anterior	Bilateral	-50.4386*	7.14	<0.01**	-67.97	-32.91
		Unilateral (right side)	-869.5906*	7.14	<0.01**	-887.12	-852.06
	Unilateral (right side)	Bilateral	819.1520*	6.81	<0.01**	802.44	835.87
		Anterior	869.5906*	7.14	<0.01**	852.06	887.12
Left MDI Vestibular SG	Bilateral	Anterior	44.0932*	7.67	<0.01**	25.27	62.92
		Unilateral (right side)	-458.0384*	7.31	<0.01**	-475.99	-440.09
	Anterior	Bilateral	-44.0932*	7.67	<0.01**	-62.92	-25.27
		Unilateral (right side)	-502.1315*	7.67	<0.01**	-520.96	-483.31
	Unilateral (right side)	Bilateral	458.0384*	7.31	<0.01**	440.09	475.99
		Anterior	502.1315*	7.67	<0.01**	483.31	520.96
Right MDI Oral SG	Bilateral	Anterior	32.6014*	7.41	<0.01**	14.41	50.79
		Unilateral (right side)	-839.0149*	7.06	<0.01**	-856.36	-821.67
	Anterior	Bilateral	-32.6014*	7.41	<0.01**	-50.79	-14.41
		Unilateral (right side)	-871.6163*	7.41	<0.01**	-889.81	-853.42
	Unilateral (right side)	Bilateral	839.0149*	7.06	<0.01**	821.67	856.36
		Anterior	871.6163*	7.41	<0.01**	853.42	889.81
Left MDI Oral SG	Bilateral	Anterior	-8.51	6.89	0.47 NS	-25.44	8.41
		Unilateral (right side)	-540.6948*	6.57	<0.01**	-556.83	-524.56
	Anterior	Bilateral	8.51	6.89	0.47 NS	-8.41	25.44
		Unilateral (right side)	-532.1806*	6.89	<0.01**	-549.11	-515.25
	Unilateral (right side)	Bilateral	540.6948*	6.57	<0.01**	524.56	556.83
		Anterior	532.1806*	6.89	<0.01**	515.25	549.11

Based on observed means.
The error term is Mean Square(Error) = 3887.793.
**. The mean difference is significant at the .05 level.*
*P=level of significance; **=significant at p<0.01; NS=not significant*

Supplementary Table 5. Descriptive Statistics: Periimplant Microstrains (Arithmetic Means and Standard Deviations) registered in the three-mini-implant models

Strain gauge position	Force	Splinting status	Loading position	Mean	Std. Deviation	N
Right MDI Vestibular Strain gauge	50 N	Not splinted (single-units)	Bilateral	518.59	52.26	15
			Anterior	528.28	65.01	15
			Unilateral (right side)	837.55	64.14	15
		Splinted	Bilateral	370.63	45.49	15
			Anterior	377.71	72.17	15
			Unilateral (right side)	687.18	55.58	15
	100 N	Not splinted (single-units)	Bilateral	752.91	62.85	15
			Anterior	590.25	59.06	15
			Unilateral (right side)	1065.31	65.22	15
		Splinted	Bilateral	597.75	50.87	15
			Anterior	457.90	52.62	15
			Unilateral (right side)	916.91	59.01	15
	150 N	Not splinted (single-units)	Bilateral	975.10	102.88	15
			Anterior	1537.15	68.04	15
			Unilateral (right side)	1256.70	47.82	15
		Splinted	Bilateral	831.40	98.42	15
			Anterior	1383.83	82.58	15
			Unilateral (right side)	1106.68	48.19	15
	200 N	Not splinted (single-units)	Bilateral	1569.51	59.41	15
			Anterior	1587.08	67.93	15
			Unilateral (right side)	1889.48	80.16	15
		Splinted	Bilateral	1414.67	61.77	15
			Anterior	1428.21	69.55	15
			Unilateral (right side)	1740.86	53.08	15
	250 N	Not splinted (single-units)	Bilateral	1808.85	62.68	15
			Anterior	1637.60	77.03	15
			Unilateral (right side)	2104.86	77.17	15
		Splinted	Bilateral	1648.65	61.42	15
			Anterior	1514.86	58.27	15
			Unilateral (right side)	1966.79	66.60	15
	300 N	Not splinted (single-units)	Bilateral	2017.19	103.61	15
			Unilateral (right side)	2298.31	43.57	15
		Splinted	Bilateral	1879.62	98.20	15
			Unilateral (right side)	2159.95	56.08	15
Left MDI Vestibular Strain gauge	50 N	Not splinted (single-units)	Bilateral	507.88	39.44	15
			Anterior	489.48	37.39	15
			Unilateral (right side)	480.45	45.77	15
		Splinted	Bilateral	342.96	34.95	15
			Anterior	341.17	27.27	15
			Unilateral (right side)	320.43	40.59	15
	100 N	Not splinted (single-units)	Bilateral	750.56	69.31	15
			Anterior	618.72	32.87	15
			Unilateral (right side)	585.07	68.15	15
		Splinted	Bilateral	986.03	87.18	15
			Anterior	1488.43	52.27	15
			Unilateral (right side)	742.28	57.26	15
	150 N	Not splinted (single-units)	Bilateral	837.75	92.67	15
			Anterior	1336.41	62.92	15

			Unilateral (right side)	587.44	55.16	15
		Splinted	Bilateral	837.75	92.67	15
			Anterior	1336.41	62.92	15
			Unilateral (right side)	587.44	55.16	15
	200 N	Not splinted (single-units)	Bilateral	1564.30	48.25	15
			Anterior	1545.76	48.51	15
			Unilateral (right side)	1526.73	66.17	15
		Splinted	Bilateral loading	1381.06	42.75	15
			Anterior loading	1384.81	40.08	15
			Unilateral (right side)	1368.82	39.16	15
	250 N	Not splinted (single-units)	Bilateral loading	1816.46	70.13	15
			Anterior loading	1672.46	50.60	15
			Unilateral (right side)	1627.62	70.78	15
		Splinted	Bilateral loading	1643.02	66.51	15
			Anterior loading	1514.69	35.89	15
			Unilateral (right side)	1489.50	63.47	15
	300 N	Not splinted (single-units)	Bilateral loading	2031.19	98.29	15
			Unilateral (right side)	1787.88	66.78	15
		Splinted	Bilateral loading	1887.90	102.71	15
			Unilateral (right side)	1627.64	51.81	15
**Right MDI Oral Strain gauge**	50 N	Not splinted (single-units)	Bilateral	518.52	62.36	15
			Anterior loading	513.18	47.88	15
			Unilateral (right side)	780.25	62.85	15
		Splinted	Bilateral	364.54	45.75	15
			Anterior loading	375.19	31.83	15
			Unilateral (right side)	621.37	55.31	15
	100 N	Not splinted (single-units)	Bilateral	689.52	60.45	15
			Anterior loading	529.03	29.13	15
			Unilateral (right side)	947.20	56.64	15
		Splinted	Bilateral	533.07	58.04	15
			Anterior loading	379.79	8.57	15
			Unilateral (right side)	796.77	57.16	15
	150 N	Not splinted (single-units)	Bilateral	849.58	71.73	15
			Anterior loading	1527.01	52.19	15
			Unilateral (right side)	1217.31	61.33	15
		Splinted	Bilateral	696.33	54.99	15
			Anterior loading	1363.69	70.44	15
			Unilateral (right side)	1065.17	41.53	15
	200 N	Not splinted (single-units)	Bilateral	1575.51	64.34	15
			Anterior loading	1566.83	55.95	15
			Unilateral (right side)	1820.80	80.20	15
		Splinted	Bilateral	1412.63	53.76	15
			Anterior loading	1418.66	55.52	15
			Unilateral (right side)	1673.99	68.51	15
	250 N	Not splinted (single-units)	Bilateral	1745.35	65.90	15
			Anterior loading	1568.02	38.02	15
			Unilateral (right side)	1993.49	76.07	15
		Splinted	Bilateral	1575.94	67.66	15
			Anterior loading	1436.02	31.30	15
			Unilateral (right side)	1846.50	55.88	15
	300 N	Not splinted (single-units)	Bilateral	1890.31	78.97	15
			Unilateral (right side)	2273.19	60.10	15
		Splinted	Bilateral	1745.09	61.33	15
			Unilateral (right side)	2109.00	51.76	15
**Left MDI**	50 N		Bilateral	491.21	43.11	15

**Oral Strain gauge**		Not splinted (single-units)	Anterior loading	485.79	31.66	15
			Unilateral (right side)	373.68	40.01	15
		Splinted	Bilateral	348.79	43.91	15
			Anterior loading	341.52	21.49	15
			Unilateral (right side)	227.11	25.81	15
	100 N	Not splinted (single-units)	Bilateral	681.13	40.60	15
			Anterior loading	504.85	32.46	15
			Unilateral (right side)	489.56	44.71	15
		Splinted	Bilateral	535.47	35.46	15
			Anterior loading	355.20	13.02	15
			Unilateral (right side)	343.90	34.33	15
	150 N	Not splinted (single-units)	Bilateral	804.68	48.24	15
			Anterior loading	1475.37	41.80	15
			Unilateral (right side)	664.83	63.48	15
		Splinted	Bilateral	651.30	37.77	15
			Anterior loading	1345.34	48.32	15
			Unilateral (right side)	520.53	47.94	15
	200 N	Not splinted (single-units)	Bilateral	1545.04	56.83	15
			Anterior loading	1521.08	29.69	15
			Unilateral (right side)	1446.87	46.46	15
		Splinted	Bilateral	1407.11	62.09	15
			Anterior loading	1391.76	30.30	15
			Unilateral (right side)	1273.14	39.67	15
	250 N	Not splinted (single-units)	Bilateral	1721.91	49.97	15
			Anterior loading	1550.12	37.55	15
			Unilateral (right side)	1540.61	53.20	15
		Splinted	Bilateral	1586.02	37.91	15
			Anterior loading	1407.02	31.95	15
			Unilateral (right side)	1379.06	31.42	15
	300 N	Not splinted (single-units)	Bilateral	1850.07	54.70	15
			Unilateral (right side)	1717.50	89.43	15
		Splinted	Bilateral	1708.09	48.52	15
			Unilateral (right side)	1566.58	54.68	15
**Midline MDI Vestibular Strain gauge**	50 N	Not splinted (single-units)	Bilateral	437.46	29.57	15
			Anterior	685.83	63.49	15
			Unilateral (right side)	692.92	48.03	15
		Splinted	Bilateral	285.39	5.69	15
			Anterior	523.72	52.16	15
			Unilateral (right side)	542.31	35.48	15
	100 N	Not splinted (single-units)	Bilateral	481.50	67.55	15
			Anterior	865.60	64.80	15
			Unilateral (right side)	926.68	57.58	15
		Splinted	Bilateral	324.35	42.10	15
			Anterior	714.87	58.08	15
			Unilateral (right side)	779.89	51.16	15
	150 N	Not splinted (single-units)	Bilateral	688.65	44.32	15
			Anterior	1707.79	74.08	15
			Unilateral (right side)	1171.88	51.72	15
		Splinted	Bilateral	534.67	40.86	15
			Anterior	1507.08	62.54	15
			Unilateral (right side)	1021.65	47.61	15
	200 N	Not splinted (single-units)	Bilateral	1503.57	36.27	15
			Anterior	1754.27	69.20	15
			Unilateral (right side)	1733.66	55.25	15
		Splinted	Bilateral	1335.43	20.37	15

			Anterior	1566.63	58.45	15
			Unilateral (right side)	1582.88	32.69	15
	250 N	Not splinted (single-units)	Bilateral	1526.25	66.27	15
			Anterior	1902.14	57.30	15
			Unilateral (right side)	1983.25	70.82	15
		Splinted	Bilateral	1382.09	55.12	15
			Anterior	1753.83	62.80	15
			Unilateral (right side)	1847.03	62.62	15
	300 N	Not splinted (single-units)	Bilateral	1738.36	45.65	15
			Unilateral (right side)	2227.08	55.71	15
		Splinted	Bilateral	1577.35	43.85	15
			Unilateral (right side)	2075.64	39.70	15
**Midline MDI Oral Strain gauge**	50 N	Not splinted (single-units)	Bilateral	391.27	40.72	15
			Anterior	590.97	46.75	15
			Unilateral (right side)	728.06	49.07	15
		Splinted	Bilateral	232.20	22.36	15
			Anterior	440.12	26.20	15
			Unilateral (right side)	582.18	44.74	15
	100 N	Not splinted (single-units)	Bilateral	487.33	50.39	15
			Anterior	787.86	34.63	15
			Unilateral (right side)	794.62	51.91	15
		Splinted	Bilateral	346.96	34.87	15
			Anterior	642.86	25.98	15
			Unilateral (right side)	647.02	34.22	15
	150 N	Not splinted (single-units)	Bilateral	717.29	44.72	15
			Anterior	1582.46	65.10	15
			Unilateral (right side)	1045.15	74.90	15
		Splinted	Bilateral	566.43	34.38	15
			Anterior	1442.29	46.46	15
			Unilateral (right side)	904.99	65.86	15
	200 N	Not splinted (single-units)	Bilateral	1440.98	46.40	15
			Anterior	1641.74	52.43	15
			Unilateral (right side)	1775.91	51.22	15
		Splinted	Bilateral loading	1282.57	36.94	15
			Anterior loading	1492.91	48.99	15
			Unilateral (right side)	1634.58	58.47	15
	250 N	Not splinted (single-units)	Bilateral loading	1540.14	54.87	15
			Anterior loading	1842.24	34.97	15
			Unilateral (right side)	1837.20	75.14	15
		Splinted	Bilateral loading	1409.23	50.73	15
			Anterior loading	1687.75	38.74	15
			Unilateral (right side)	1693.55	44.11	15
	300 N	Not splinted (single-units)	Bilateral loading	1773.37	59.69	15
			Unilateral (right side)	2106.15	90.38	15
		Splinted	Bilateral loading	1623.46	39.38	15
			Unilateral (right side)	1960.57	60.14	15

Supplementary Table 6. Multivariate analysis (tests of between-subjects effects) of periimplant microstrains (dependent variables) registered in the three mini-implant models with mini-implants having different splinting status (single-units or splinted) loaded with forces varying from 50 to 300 N at different loading positions (bilateral, anterior, unilateral-right side)

Tests of Between-Subjects Effects

Source	Strain gauge position	Type III Sum of Squares	df	Mean Square	F	*P*	Partial Eta Squared
Corrected Model	Right Vestibular SG	165654668.238[a]	33	5019838.43	1084.88	<0.01**	0.99
	Left Vestibular SG	150575151.052[b]	33	4562883.37	1306.10	<0.01**	0.99
	Right Oral SG	161611929.960[c]	33	4897331.21	1473.97	<0.01**	0.99
	Left Oral SG	147225708.025[d]	33	4461385.09	2224.88	<0.01**	0.99
	Midline Vestibular SG	164179745.219[e]	33	4975143.79	1772.14	<0.01**	0.99
	Midline Oral SG	156464636.407[f]	33	4741352.62	1873.11	<0.01**	0.99
Intercept	Right Vestibular SG	848647980.59	1	848647980.59	183408.10	<0.01**	1.00
	Left Vestibular SG	651350292.11	1	651350292.11	186445.36	<0.01**	1.00
	Right Oral SG	772775302.44	1	772775302.44	232584.65	<0.01**	1.00
	Left Oral SG	568496925.17	1	568496925.17	283508.39	<0.01**	1.00
	Midline Vestibular SG	783963747.31	1	783963747.31	279246.93	<0.01**	1.00
	Midline Oral SG	720428806.56	1	720428806.56	284611.67	<0.01**	1.00
Force	Right Vestibular SG	141057081.70	5	28211416.34	6096.99	<0.01**	0.98
	Left Vestibular SG	137686843.64	5	27537368.73	7882.42	<0.01**	0.99
	Right Oral SG	137513722.33	5	27502744.47	8277.59	<0.01**	0.99
	Left Oral SG	132550730.90	5	26510146.18	13220.56	<0.01**	0.99
	Midline Vestibular SG	138651424.45	5	27730284.89	9877.49	<0.01**	0.99
	Midline Oral SG	137678029.18	5	27535605.84	10878.18	<0.01**	0.99
Splinting status	Right Vestibular SG	2664828.86	1	2664828.86	575.92	<0.01**	0.55
	Left Vestibular SG	3036719.62	1	3036719.62	869.24	<0.01**	0.65
	Right Oral SG	2894442.36	1	2894442.36	871.15	<0.01**	0.65
	Left Oral SG	2640240.78	1	2640240.78	1316.68	<0.01**	0.73
	Midline Vestibular SG	3118836.64	1	3118836.64	1110.93	<0.01**	0.70
	Midline Oral SG	2693889.14	1	2693889.14	1064.24	<0.01**	0.69
Loading position	Right Vestibular SG	9266697.63	2	4633348.82	1001.35	<0.01**	0.81
	Left Vestibular SG	3084607.79	2	1542303.89	441.48	<0.01**	0.65
	Right Oral SG	8322345.01	2	4161172.50	1252.40	<0.01**	0.84
	Left Oral SG	3851239.45	2	1925619.72	960.30	<0.01**	0.80
	Midline Vestibular SG	20845974.71	2	10422987.35	3712.66	<0.01**	0.94
	Midline Oral SG	13946498.42	2	6973249.21	2754.84	<0.01**	0.92
*Force * Splinting status*	Right Vestibular SG	4129.17	5	825.83	0.18	0.97 NS	0.00
	Left Vestibular SG	4538.82	5	907.76	0.26	0.93 NS	0.00

	Right Oral SG	624.30	5	124.86	0.04	1.00 NS	0.00
	Left Oral SG	422.29	5	84.46	0.04	1.00 NS	0.00
	Midline Vestibular SG	11591.42	5	2318.28	0.83	0.53 NS	0.01
	Midline Oral SG	1466.84	5	293.37	0.12	0.99 NS	0.00
*Force * Loading positiion*	Right Vestibular SG	7208219.87	9	800913.32	173.09	<0.01**	0.77
	Left Vestibular SG	7550815.74	9	838979.53	240.15	<0.01**	0.82
	Right Oral SG	8507745.11	9	945305.01	284.51	<0.01**	0.84
	Left Oral SG	9823351.32	9	1091483.48	544.32	<0.01**	0.91
	Midline Vestibular SG	7164564.50	9	796062.72	283.56	<0.01**	0.84
	Midline Oral SG	6333758.54	9	703750.95	278.02	<0.01**	0.84
*Splinting status * Loading position*	Right Vestibular SG	1353.85	2	676.93	0.15	0.86 NS	0.00
	Left Vestibular SG	1105.54	2	552.77	0.16	0.85 NS	0.00
	Right Oral SG	2305.00	2	1152.50	0.35	0.71 NS	0.00
	Left Oral SG	4903.40	2	2451.70	1.22	0.30 NS	0.01
	Midline Vestibular SG	10504.06	2	5252.03	1.87	0.16 NS	0.01
	Midline Oral SG	504.58	2	252.29	0.10	0.91 NS	0.00
*Force * Splinting status * Loading position*	Right Vestibular SG	6804.28	9	756.03	0.16	1.00 NS	0.00
	Left Vestibular SG	9061.76	9	1006.86	0.29	0.98 NS	0.01
	Right Oral SG	8132.06	9	903.56	0.27	0.98 NS	0.01
	Left Oral SG	8566.07	9	951.79	0.47	0.89 NS	0.01
	Midline Vestibular SG	8346.60	9	927.40	0.33	0.96 NS	0.01
	Midline Oral SG	4185.69	9	465.08	0.18	1.00 NS	0.00
Error	Right Vestibular SG	2197873.40	475	4627.10			
	Left Vestibular SG	1659421.26	475	3493.52			
	Right Oral SG	1578213.67	475	3322.56			
	Left Oral SG	952479.89	475	2005.22			
	Midline Vestibular SG	1333525.05	475	2807.42			
	Midline Oral SG	1202352.97	475	2531.27			
Total	Right Vestibular SG	1001175776.37	509				
	Left Vestibular SG	782122293.63	509				
	Right Oral SG	920223579.75	509				
	Left Oral SG	696402696.26	509				
	Midline Vestibular SG	921697340.52	509				
	Midline Oral SG	852588560.74	509				
Corrected Total	Right Vestibular SG	167852541.64	508				
	Left Vestibular SG	152234572.31	508				
	Right Oral SG	163190143.63	508				
	Left Oral SG	148178187.92	508				
	Midline Vestibular SG	165513270.27	508				
	Midline Oral SG	157666989.38	508				

a. R Squared = .987 (Adjusted R Squared = .986)
b. R Squared = .989 (Adjusted R Squared = .988)
c. R Squared = .990 (Adjusted R Squared = .990)
d. R Squared = .994 (Adjusted R Squared = .993)
e. R Squared = .992 (Adjusted R Squared = .991)

f. R Squared = .992 (Adjusted R Squared = .992)

**=p<0.01; NS=not significant; *P*=Level of significance; df=degree of freedom; SG= strain gauge

Supplementary Table 7. Multiple comparisons: post-hoc tests (Sheffe): Significance of the differences of periimplant microstrains in the three-MDI Models during the mandibular overdenture loading under different forces (50, 100, 150, 200, 250, and 300 N, respectively

Multiple comparisons: Sheffe

**Right MDI Vestibular SG**	50 N	100 N	-178.3355*	<0.001	-212.31	-144.36
		150 N	-628.4873*	<0.001	-662.37	-594.60
		200 N	-1051.6476*	<0.001	-1085.53	-1017.77
		250 N	-1226.9450*	<0.001	-1260.83	-1193.06
		300 N	-1535.4464*	<0.001	-1573.33	-1497.56
	100 N	50 N	178.3355*	<0.001	144.36	212.31
		150 N	-450.1518*	<0.001	-484.13	-416.17
		200 N	-873.3121*	<0.001	-907.29	-839.33
		250 N	-1048.6095*	<0.001	-1082.59	-1014.63
		300 N	-1357.1109*	<0.001	-1395.08	-1319.14
	150 N	50 N	628.4873*	<0.001	594.60	662.37
		100 N	450.1518*	<0.001	416.17	484.13
		200 N	-423.1603*	<0.001	-457.04	-389.28
		250 N	-598.4577*	<0.001	-632.34	-564.58
		300 N	-906.9591*	<0.001	-944.84	-869.08
	200 N	50 N	1051.6476*	<0.001	1017.77	1085.53
		100 N	873.3121*	<0.001	839.33	907.29
		150 N	423.1603*	<0.001	389.28	457.04
		250 N	-175.2974*	<0.001	-209.18	-141.41
		300 N	-483.7988*	<0.001	-521.68	-445.92
	250 N	50 N	1226.9450*	<0.001	1193.06	1260.83
		100 N	1048.6095*	<0.001	1014.63	1082.59
		150 N	598.4577*	<0.001	564.58	632.34
		200 N	175.2974*	<0.001	141.41	209.18
		300 N	-308.5014*	<0.001	-346.38	-270.62
	300 N	50 N	1535.4464*	<0.001	1497.56	1573.33
		100 N	1357.1109*	<0.001	1319.14	1395.08
		150 N	906.9591*	<0.001	869.08	944.84
		200 N	483.7988*	<0.001	445.92	521.68
		250 N	308.5014*	<0.001	270.62	346.38
**Left MDI Vestibular SG**	50 N	100 N	-162.3222*	<0.001	-191.85	-132.80
		150 N	-582.6600*	<0.001	-612.10	-553.22
		200 N	-1048.1827*	<0.001	-1077.62	-1018.74
		250 N	-1213.5627*	<0.001	-1243.00	-1184.12
		300 N	-1419.9198*	<0.001	-1452.84	-1387.00
	100 N	50 N	162.3222*	<0.001	132.80	191.85
		150 N	-420.3379*	<0.001	-449.86	-390.81
		200 N	-885.8605*	<0.001	-915.38	-856.34
		250 N	-1051.2405*	<0.001	-1080.76	-1021.72
		300 N	-1257.5977*	<0.001	-1290.59	-1224.61
	150 N	50 N	582.6600*	<0.001	553.22	612.10
		100 N	420.3379*	<0.001	390.81	449.86
		200 N	-465.5227*	<0.001	-494.96	-436.08
		250 N	-630.9027*	<0.001	-660.34	-601.46
		300 N	-837.2598*	<0.001	-870.18	-804.34

	200 N	50 N	1048.1827*	<0.001	1018.74	1077.62
		100 N	885.8605*	<0.001	856.34	915.38
		150 N	465.5227*	<0.001	436.08	494.96
		250 N	-165.3800*	<0.001	-194.82	-135.94
		300 N	-371.7371*	<0.001	-404.65	-338.82
	250 N	50 N	1213.5627*	<0.001	1184.12	1243.00
		100 N	1051.2405*	<0.001	1021.72	1080.76
		150 N	630.9027*	<0.001	601.46	660.34
		200 N	165.3800*	<0.001	135.94	194.82
		300 N	-206.3571*	<0.001	-239.27	-173.44
	300 N	50 N	1419.9198*	<0.001	1387.00	1452.84
		100 N	1257.5977*	<0.001	1224.61	1290.59
		150 N	837.2598*	<0.001	804.34	870.18
		200 N	371.7371*	<0.001	338.82	404.65
		250 N	206.3571*	<0.001	173.44	239.27
**Right MDI Oral SG**	50 N	100 N	-118.3223*	<0.001	-147.11	-89.53
		150 N	-591.0061*	<0.001	-619.72	-562.29
		200 N	-1049.2270*	<0.001	-1077.94	-1020.52
		250 N	-1165.3783*	<0.001	-1194.09	-1136.67
		300 N	-1475.5563*	<0.001	-1507.66	-1443.46
	100 N	50 N	118.3223*	<0.001	89.53	147.11
		150 N	-472.6839*	<0.001	-501.48	-443.89
		200 N	-930.9047*	<0.001	-959.70	-902.11
		250 N	-1047.0560*	<0.001	-1075.85	-1018.26
		300 N	-1357.2340*	<0.001	-1389.41	-1325.06
	150 N	50 N	591.0061*	<0.001	562.29	619.72
		100 N	472.6839*	<0.001	443.89	501.48
		200 N	-458.2208*	<0.001	-486.93	-429.51
		250 N	-574.3722*	<0.001	-603.08	-545.66
		300 N	-884.5501*	<0.001	-916.65	-852.45
	200 N	50 N	1049.2270*	<0.001	1020.52	1077.94
		100 N	930.9047*	<0.001	902.11	959.70
		150 N	458.2208*	<0.001	429.51	486.93
		250 N	-116.1513*	<0.001	-144.86	-87.44
		300 N	-426.3293*	<0.001	-458.43	-394.23
	250 N	50 N	1165.3783*	<0.001	1136.67	1194.09
		100 N	1047.0560*	<0.001	1018.26	1075.85
		150 N	574.3722*	<0.001	545.66	603.08
		200 N	116.1513*	<0.001	87.44	144.86
		300 N	-310.1780*	<0.001	-342.28	-278.08
	300 N	50 N	1475.5563*	<0.001	1443.46	1507.66
		100 N	1357.2340*	<0.001	1325.06	1389.41
		150 N	884.5501*	<0.001	852.45	916.65
		200 N	426.3293*	<0.001	394.23	458.43
		250 N	310.1780*	<0.001	278.08	342.28
**Left Oral SG**	50 N	100 N	-106.4347*	<0.001	-128.80	-84.07
		150 N	-532.3233*	<0.001	-554.63	-510.02
		200 N	-1052.8163*	<0.001	-1075.12	-1030.51
		250 N	-1152.7732*	<0.001	-1175.08	-1130.47
		300 N	-1332.5412*	<0.001	-1357.48	-1307.60
	100 N	50 N	106.4347*	<0.001	84.07	128.80
		150 N	-425.8886*	<0.001	-448.26	-403.52
		200 N	-946.3817*	<0.001	-968.75	-924.01
		250 N	-1046.3386*	<0.001	-1068.71	-1023.97
		300 N	-1226.1065*	<0.001	-1251.10	-1201.11

	150 N	50 N	532.3233*	<0.001	510.02	554.63
		100 N	425.8886*	<0.001	403.52	448.26
		200 N	-520.4931*	<0.001	-542.80	-498.19
		250 N	-620.4499*	<0.001	-642.76	-598.14
		300 N	-800.2179*	<0.001	-825.16	-775.28
	200 N	50 N	1052.8163*	<0.001	1030.51	1075.12
		100 N	946.3817*	<0.001	924.01	968.75
		150 N	520.4931*	<0.001	498.19	542.80
		250 N	-99.9569*	<0.001	-122.26	-77.65
		300 N	-279.7249*	<0.001	-304.66	-254.79
	250 N	50 N	1152.7732*	<0.001	1130.47	1175.08
		100 N	1046.3386*	<0.001	1023.97	1068.71
		150 N	620.4499*	<0.001	598.14	642.76
		200 N	99.9569*	<0.001	77.65	122.26
		300 N	-179.7680*	<0.001	-204.71	-154.83
	300 N	50 N	1332.5412*	<0.001	1307.60	1357.48
		100 N	1226.1065*	<0.001	1201.11	1251.10
		150 N	800.2179*	<0.001	775.28	825.16
		200 N	279.7249*	<0.001	254.79	304.66
		250 N	179.7680*	<0.001	154.83	204.71
**Midline MDI Vestibular SG**	50 N	100 N	-158.2328*	<0.001	-184.70	-131.77
		150 N	-577.3513*	<0.001	-603.74	-550.96
		200 N	-1051.4719*	<0.001	-1077.86	-1025.08
		250 N	-1204.4961*	<0.001	-1230.89	-1178.10
		300 N	-1376.6691*	<0.001	-1406.18	-1347.16
	100 N	50 N	158.2328*	<0.001	131.77	184.70
		150 N	-419.1185*	<0.001	-445.58	-392.65
		200 N	-893.2391*	<0.001	-919.71	-866.77
		250 N	-1046.2632*	<0.001	-1072.73	-1019.80
		300 N	-1218.4363*	<0.001	-1248.01	-1188.86
	150 N	50 N	577.3513*	<0.001	550.96	603.74
		100 N	419.1185*	<0.001	392.65	445.58
		200 N	-474.1206*	<0.001	-500.51	-447.73
		250 N	-627.1448*	<0.001	-653.54	-600.75
		300 N	-799.3178*	<0.001	-828.83	-769.81
	200 N	50 N	1051.4719*	<0.001	1025.08	1077.86
		100 N	893.2391*	<0.001	866.77	919.71
		150 N	474.1206*	<0.001	447.73	500.51
		250 N	-153.0242*	<0.001	-179.42	-126.63
		300 N	-325.1972*	<0.001	-354.70	-295.69
	250 N	50 N	1204.4961*	<0.001	1178.10	1230.89
		100 N	1046.2632*	<0.001	1019.80	1072.73
		150 N	627.1448*	<0.001	600.75	653.54
		200 N	153.0242*	<0.001	126.63	179.42
		300 N	-172.1730*	<0.001	-201.68	-142.67
	300 N	50 N	1376.6691*	<0.001	1347.16	1406.18
		100 N	1218.4363*	<0.001	1188.86	1248.01
		150 N	799.3178*	<0.001	769.81	828.83
		200 N	325.1972*	<0.001	295.69	354.70
		250 N	172.1730*	<0.001	142.67	201.68
**Midline MDI Oral SG**	50 N	100 N	-126.6830*	<0.001	-151.81	-101.55
		150 N	-548.9675*	<0.001	-574.03	-523.91
		200 N	-1050.6477*	<0.001	-1075.71	-1025.59
		250 N	-1174.2166*	<0.001	-1199.28	-1149.16
		300 N	-1371.7546*	<0.001	-1399.77	-1343.74

	100 N	50 N	126.6830*	<0.001	101.55	151.81
		150 N	-422.2845*	<0.001	-447.42	-397.15
		200 N	-923.9648*	<0.001	-949.10	-898.83
		250 N	-1047.5337*	<0.001	-1072.66	-1022.40
		300 N	-1245.0716*	<0.001	-1273.15	-1216.99
	150 N	50 N	548.9675*	<0.001	523.91	574.03
		100 N	422.2845*	<0.001	397.15	447.42
		200 N	-501.6803*	<0.001	-526.74	-476.62
		250 N	-625.2492*	<0.001	-650.31	-600.19
		300 N	-822.7871*	<0.001	-850.81	-794.77
	200 N	50 N	1050.6477*	<0.001	1025.59	1075.71
		100 N	923.9648*	<0.001	898.83	949.10
		150 N	501.6803*	<0.001	476.62	526.74
		250 N	-123.5689*	<0.001	-148.63	-98.51
		300 N	-321.1069*	<0.001	-349.13	-293.09
	250 N	50 N	1174.2166*	<0.001	1149.16	1199.28
		100 N	1047.5337*	<0.001	1022.40	1072.66
		150 N	625.2492*	<0.001	600.19	650.31
		200 N	123.5689*	<0.001	98.51	148.63
		300 N	-197.5380*	<0.001	-225.56	-169.52
	300 N	50 N	1371.7546*	<0.001	1343.74	1399.77
		100 N	1245.0716*	<0.001	1216.99	1273.15
		150 N	822.7871*	<0.001	794.77	850.81
		200 N	321.1069*	<0.001	293.09	349.13
		250 N	197.5380*	<0.001	169.52	225.56

P=p value; *. The mean difference is significant at the .05 level;

Supplementary Table 8. Multiple comparisons: post-hoc tests (Sheffe): Significance of the differences of periimplant microstrains in the three-MDI Models during the mandibular overdenture loading at different loading positions (Bilaterally, anteriorly and unilaterally-right side)

Multiple Comparisons: Scheffe						
**Strain gauge position**	**Loading position**		**Mean Difference (I-J)**	***P***	**95% Confidence Interval**	
					**Lower Bound**	**Upper Bound**
**Right MDI Vestibular SG**	Bilateral loading	Anterior loading	97.8087*	<0.01	79.32	116.30
		Unilateral loading (right side)	-300.4522*	<0.01	-318.08	-282.82
	Anterior loading	Bilateral loading	-97.8087*	.<0.01	-116.30	-79.32
		Unilateral loading (right side)	-398.2608*	.<0.01	-416.73	-379.80
	Unilateral loading (right side)	Bilateral loading	300.4522*	<0.01	282.82	318.08
		Anterior loading	398.2608*	<0.01	379.80	416.73
**Left MDI Vestibular SG**	Bilateral loading	Anterior loading	113.8511*	.<0.01	97.79	129.92
		Unilateral loading (right side)	151.2148*	.<0.01	135.89	166.53
	Anterior loading	Bilateral loading	-113.8511*	<0.01	-129.92	-97.79
		Unilateral loading (right side)	37.3636*	<0.01	21.32	53.41
	Unilateral loading (right side)	Bilateral loading	-151.2148*	.<0.01	-166.53	-135.89
		Anterior loading	-37.3636*	.<0.01	-53.41	-21.32
**Right MDI Oral SG**	Bilateral loading	Anterior loading	68.6415*	<0.01	52.97	84.31
		Unilateral loading (right side)	-292.3679*	<0.01	-307.31	-277.43
	Anterior loading	Bilateral loading	-68.6415*	.<0.01	-84.31	-52.97
		Unilateral loading (right side)	-361.0094*	.<0.01	-376.66	-345.36
	Unilateral loading (right side)	Bilateral loading	292.3679*	<0.01	277.43	307.31
		Anterior loading	361.0094*	<0.01	345.36	376.66
**Left MDI Oral SG**	Bilateral loading	Anterior loading	76.3112*	.<0.01	64.14	88.48
		Unilateral loading (right side)	152.1698*	.<0.01	140.56	163.78
	Anterior loading	Bilateral loading	-76.3112*	<0.01	-88.48	-64.14
		Unilateral loading (right side)	75.8585*	<0.01	63.70	88.01
	Unilateral loading (right side)	Bilateral loading	-152.1698*	.<0.01	-163.78	-140.56
		Anterior loading	-75.8585*	.<0.01	-88.01	-63.70
**Midline MDI Vestibular SG**	Bilateral loading	Anterior loading	-309.8981*	<0.01	-324.30	-295.50
		Unilateral loading (right side)	-393.7963*	<0.01	-407.53	-380.06
	Anterior loading	Bilateral loading	309.8981*	.<0.01	295.50	324.30
		Unilateral loading (right side)	-83.8982*	.<0.01	-98.28	-69.51
	Unilateral loading (right side)	Bilateral loading	393.7963*	<0.01	380.06	407.53
		Anterior loading	83.8982*	<0.01	69.51	98.28
**Midline MDI Oral SG**	Bilateral loading	Anterior loading	-227.2881*	.<0.01	-240.96	-213.61
		Unilateral loading (right side)	-321.3355*	.<0.01	-334.38	-308.30

Anterior loading	Bilateral loading	227.2881*	<0.01	213.61	240.96
	Unilateral loading (right side)	-94.0473*	<0.01	-107.71	-80.39
Unilateral loading (right side)	Bilateral loading	321.3355*	.<0.01	308.30	334.38
	Anterior loading	94.0473*	.<0.01	80.39	107.71

Based on observed means.
The error term is Mean Square(Error) = 2531.269.
***. The mean difference is significant at the .05 level.; *P* = p value**

Supplementary Table 9. Means and standard deviations of periimplant microstrains registered in the four-MDI models with different splinting status (single-unit or splinted MDIs) while the respective ODs were loaded at different loading positions (bilaterally, anteriorly and unilaterally-right side) with forces varying from 50 to 300 N.

Descriptive Statistics

**Strain Gauge position**	**Force**	**Splinting status**	**Loading position**	**Mean**	**Standard Deviation**	**N**
**Right posterior MDI Vestibular SG**	50 N	Not splinted (single-units)	Bilateral	473.25	83.98	15
			Anterior	402.72	41.17	15
			Unilateral (right side)	603.54	81.27	15
		Splinted	Bilateral	333.39	29.03	15
			Anterior	215.34	5.72	15
			Unilateral (right side)	508.72	52.43	15
	100 N	Not splinted (single-units)	Bilateral	677.70	74.17	15
			Anterior	485.08	44.68	15
			Unilateral (right side)	793.13	41.78	15
		Splinted	Bilateral	537.98	42.02	15
			Anterior	434.34	48.82	15
			Unilateral (right side)	708.33	65.94	15
	150 N	Not splinted (single-units)	Bilateral	848.72	61.22	15
			Anterior	1420.18	64.93	15
			Unilateral (right side)	989.64	63.08	15
		Splinted	Bilateral	754.37	51.20	15
			Anterior	1204.71	49.87	15
			Unilateral (right side)	884.15	84.17	15
	200 N	Not splinted (single-units)	Bilateral	1516.62	93.39	15
			Anterior	1465.63	51.79	15
			Unilateral (right side)	1656.64	74.86	15
		Splinted	Bilateral	1394.51	31.72	15
			Anterior	1251.42	35.12	15
			Unilateral (right side)	1550.49	60.89	15
	250 N	Not splinted (single-units)	Bilateral	1730.65	73.61	15
			Anterior	1523.53	38.10	15
			Unilateral (right side)	1845.55	37.52	15
		Splinted	Bilateral	1582.59	54.28	15
			Anterior	1480.67	75.71	15
			Unilateral (right side)	1736.73	69.45	15
	300 N	Not splinted (single-units)	Bilateral	1895.20	65.61	15
			Unilateral (right side)	2037.30	75.19	15
		Splinted	Bilateral	1816.56	55.43	15

			Unilateral (right side)	1930.76	97.44	15
Left Posterior MDI Vestibular SG	50 N	Not splinted (single-units)	Bilateral	413.11	50.68	15
			Anterior	434.74	35.86	15
			Unilateral (right side)	304.33	34.03	15
		Splinted	Bilateral	347.00	24.37	15
			Anterior	228.02	13.93	15
			Unilateral (right side)	145.05	25.55	15
	100 N	Not splinted (single-units)	Bilateral	723.16	67.40	15
			Anterior	491.69	44.42	15
			Unilateral (right side)	427.27	52.18	15
		Splinted	Bilateral	530.58	43.56	15
			Anterior	459.11	22.90	15
			Unilateral (right side)	243.02	19.40	15
	150 N	Not splinted (single-units)	Bilateral	864.48	49.74	15
			Anterior	1449.96	49.46	15
			Unilateral (right side)	458.80	28.42	15
		Splinted	Bilateral	786.83	43.39	15
			Anterior	1230.97	34.60	15
			Unilateral (right side)	452.74	34.38	15
	200 N	Not splinted (single-units)	Bilateral	1464.00	50.72	15
			Anterior	1486.06	41.64	15
			Unilateral (right side)	1365.44	45.81	15
		Splinted	Bilateral	1391.84	42.52	15
			Anterior	1280.84	29.32	15
			Unilateral (right side)	1197.29	38.90	15
	250 N	Not splinted (single-units)	Bilateral	1777.33	80.31	15
			Anterior	1526.38	55.53	15
			Unilateral (right side)	1462.10	70.62	15
		Splinted	Bilateral	1572.07	40.96	15
			Anterior	1490.00	41.24	15
			Unilateral (right side)	1298.32	34.27	15
	300 N	Not splinted (single-units)	Bilateral	1918.68	53.39	15
			Unilateral (right side)	1517.67	40.03	15
		Splinted	Bilateral	1829.47	45.81	15
			Unilateral (right side)	1507.27	58.24	15
Right Posterior MDI Oral SG	50 N	Not splinted (single-units)	Bilateral	394.63	70.15	15
			Anterior	268.74	34.99	15
			Unilateral (right side)	529.77	77.02	15
		Splinted	Bilateral	263.83	32.45	15
			Anterior	167.21	9.71	15
			Unilateral (right side)	505.57	47.75	15
	100 N	Not splinted (single-units)	Bilateral	583.32	49.45	15
			Anterior	419.18	51.03	15
			Unilateral (right side)	695.88	59.33	15
		Splinted	Bilateral	484.71	56.74	15
			Anterior	263.92	38.47	15
			Unilateral (right side)	671.02	33.33	15
	150 N	Not splinted (single-units)	Bilateral	803.51	40.72	15
			Anterior	1268.39	50.83	15
			Unilateral (right side)	904.48	54.12	15
		Splinted	Bilateral	639.18	39.69	15
			Anterior	1192.85	35.67	15
			Unilateral (right side)	703.42	86.65	15
	200 N	Not splinted (single-units)	Bilateral	1434.11	61.15	15
			Anterior	1318.94	44.80	15
			Unilateral (right side)	1581.43	96.12	15

		Splinted	Bilateral	1306.48	44.44	15
			Anterior	1223.70	31.30	15
			Unilateral (right side)	1552.12	56.55	15
	250 N	Not splinted (single-units)	Bilateral	1639.10	62.62	15
			Anterior	1462.94	59.93	15
			Unilateral (right side)	1743.19	66.19	15
		Splinted	Bilateral	1547.93	66.53	15
			Anterior	1313.99	44.39	15
			Unilateral (right side)	1723.88	53.10	15
	300 N	Not splinted (single-units)	Bilateral	1841.35	48.52	15
			Unilateral (right side)	1954.43	57.22	15
		Splinted	Bilateral	1687.31	42.92	15
			Unilateral (right side)	1747.32	83.50	15
**Left Posterior MDI Oral SG**	50 N	Not splinted (single-units)	Bilateral	386.54	37.05	15
			Anterior	303.89	36.11	15
			Unilateral (right side)	305.71	25.90	15
		Splinted	Bilateral	283.70	41.72	15
			Anterior	162.88	16.87	15
			Unilateral (right side)	160.15	15.49	15
	100 N	Not splinted (single-units)	Bilateral	576.33	46.51	15
			Anterior	393.88	39.22	15
			Unilateral (right side)	387.17	56.51	15
		Splinted	Bilateral	503.78	25.82	15
			Anterior	376.99	44.91	15
			Unilateral (right side)	253.69	32.34	15
	150 N	Not splinted (single-units)	Bilateral	790.48	31.02	15
			Anterior	1310.28	43.94	15
			Unilateral (right side)	546.38	72.24	15
		Splinted	Bilateral	583.17	53.07	15
			Anterior	1006.03	26.05	15
			Unilateral (right side)	315.05	52.57	15
	200 N	Not splinted (single-units)	Bilateral	1442.83	43.83	15
			Anterior	1352.38	42.29	15
			Unilateral (right side)	1358.79	34.38	15
		Splinted	Bilateral	1050.16	35.29	15
			Anterior	1061.65	19.19	15
			Unilateral (right side)	1048.91	27.07	15
	250 N	Not splinted (single-units)	Bilateral	1619.48	48.46	15
			Anterior	1457.72	40.12	15
			Unilateral (right side)	1434.74	69.81	15
		Splinted	Bilateral	1048.00	25.55	15
			Anterior	1051.24	31.22	15
			Unilateral (right side)	1068.18	21.85	15
	300 N	Not splinted (single-units)	Bilateral	1844.77	34.72	15
			Unilateral (right side)	1589.53	80.68	15
		Splinted	Bilateral	1052.07	27.99	15
			Unilateral (right side)	1054.20	33.47	15
**Right Anterior MDI Vestibular SG**	50 N	Not splinted (single-units)	Bilateral	393.27	57.90	15
			Anterior	518.45	44.32	15
			Unilateral (right side)	500.58	31.07	15
		Splinted	Bilateral	250.71	34.10	15
			Anterior	397.58	70.00	15
			Unilateral (right side)	328.49	25.36	15
	100 N	Not splinted (single-units)	Bilateral	392.59	58.66	15
			Anterior	624.88	57.56	15
			Unilateral (right side)	691.10	56.10	15

		Splinted	Bilateral	343.76	27.01	15
			Anterior	614.08	18.01	15
			Unilateral (right side)	497.89	47.77	15
	150 N	Not splinted (single-units)	Bilateral	638.56	61.63	15
			Anterior	1534.21	47.95	15
			Unilateral (right side)	805.30	31.86	15
		Splinted	Bilateral	515.94	54.16	15
			Anterior	1402.22	72.16	15
			Unilateral (right side)	648.19	29.55	15
	200 N	Not splinted (single-units)	Bilateral	1378.17	53.36	15
			Anterior	1573.38	49.04	15
			Unilateral (right side)	1538.48	45.00	15
		Splinted	Bilateral	1304.53	47.25	15
			Anterior	1453.47	77.98	15
			Unilateral (right side)	1363.67	39.69	15
	250 N	Not splinted (single-units)	Bilateral	1442.31	65.28	15
			Anterior	1669.56	60.69	15
			Unilateral (right side)	1745.34	57.18	15
		Splinted	Bilateral	1386.66	44.34	15
			Anterior	1655.46	36.22	15
			Unilateral (right side)	1569.24	53.97	15
	300 N	Not splinted (single-units)	Bilateral	1684.26	57.97	15
			Unilateral (right side)	1849.58	48.18	15
		Splinted	Bilateral	1548.24	58.18	15
			Unilateral (right side)	1692.64	40.68	15
**Left Anterior MDI Vestibular l SG**	50 N	Not splinted (single-units)	Bilateral	284.89	38.62	15
			Anterior	490.11	32.97	15
			Unilateral (right side)	400.39	42.72	15
		Splinted	Bilateral	247.48	29.22	15
			Anterior	430.29	10.47	15
			Unilateral (right side)	235.94	23.32	15
	100 N	Not splinted (single-units)	Bilateral	413.32	55.07	15
			Anterior	611.32	56.61	15
			Unilateral (right side)	494.16	72.07	15
		Splinted	Bilateral	351.94	31.60	15
			Anterior	614.46	52.91	15
			Unilateral (right side)	308.32	37.22	15
	150 N	Not splinted (single-units)	Bilateral	645.28	43.36	15
			Anterior	1508.15	54.80	15
			Unilateral (right side)	606.80	59.34	15
		Splinted	Bilateral	513.13	55.46	15
			Anterior	1415.11	47.98	15
			Unilateral (right side)	530.62	30.57	15
	200 N	Not splinted (single-units)	Bilateral	1334.51	49.16	15
			Anterior	1554.66	41.16	15
			Unilateral (right side)	1449.12	59.33	15
		Splinted	Bilateral	1287.38	37.67	15
			Anterior	1468.75	37.09	15
			Unilateral (right side)	1290.22	33.62	15
	250 N	Not splinted (single-units)	Bilateral	1462.21	62.93	15
			Anterior	1654.58	65.80	15
			Unilateral (right side)	1558.79	68.86	15
		Splinted	Bilateral	1405.78	53.47	15
			Anterior	1665.86	56.95	15
			Unilateral (right side)	1367.17	50.29	15
	300 N	Not splinted	Bilateral	1699.85	49.29	15

		(single-units)	Unilateral (right side)	1657.03	64.37	15
		Splinted	Bilateral	1568.58	67.73	15
			Unilateral (right side)	1573.60	40.24	15
**Right Anterior MDI Oral SG**	50 N	Not splinted (single-units)	Bilateral	296.57	45.76	15
			Anterior	387.69	51.89	15
			Unilateral (right side)	481.10	57.13	15
		Splinted	Bilateral	134.91	19.82	15
			Anterior	252.35	23.41	15
			Unilateral (right side)	342.15	50.15	15
	100 N	Not splinted (single-units)	Bilateral	377.74	32.23	15
			Anterior	500.19	57.17	15
			Unilateral (right side)	609.00	38.59	15
		Splinted	Bilateral	252.85	31.31	15
			Anterior	551.55	28.92	15
			Unilateral (right side)	464.64	50.06	15
	150 N	Not splinted (single-units)	Bilateral	603.45	45.85	15
			Anterior	1374.73	69.78	15
			Unilateral (right side)	709.65	54.90	15
		Splinted	Bilateral	332.89	79.48	15
			Anterior	1246.41	60.93	15
			Unilateral (right side)	563.25	50.08	15
	200 N	Not splinted (single-units)	Bilateral	1346.93	58.11	15
			Anterior	1429.87	61.18	15
			Unilateral (right side)	1519.88	72.41	15
		Splinted	Bilateral	1181.73	27.65	15
			Anterior	1297.83	37.62	15
			Unilateral (right side)	1392.63	61.27	15
	250 N	Not splinted (single-units)	Bilateral	1430.16	42.66	15
			Anterior	1548.93	50.57	15
			Unilateral (right side)	1653.18	50.90	15
		Splinted	Bilateral	1301.08	40.85	15
			Anterior	1604.42	52.56	15
			Unilateral (right side)	1518.42	43.05	15
	300 N	Not splinted (single-units)	Bilateral	1669.84	61.88	15
			Unilateral (right side)	1762.47	71.90	15
		Splinted	Bilateral	1384.87	96.63	15
			Unilateral (right side)	1608.02	48.05	15
**Left Anterior MDI Oral SG**	50 N	Not splinted (single-units)	Bilateral	314.14	35.72	15
			Anterior	380.28	40.34	15
			Unilateral (right side)	413.90	41.61	15
		Splinted	Bilateral	165.28	20.38	15
			Anterior	253.47	28.51	15
			Unilateral (right side)	245.31	22.74	15
	100 N	Not splinted (single-units)	Bilateral	410.17	71.29	15
			Anterior	501.06	39.01	15
			Unilateral (right side)	494.59	33.30	15
		Splinted	Bilateral	237.89	15.79	15
			Anterior	565.50	44.10	15
			Unilateral (right side)	337.72	23.88	15
	150 N	Not splinted (single-units)	Bilateral	606.92	48.84	15
			Anterior	1392.09	55.07	15
			Unilateral (right side)	599.60	84.99	15
		Splinted	Bilateral	410.69	23.60	15
			Anterior	1265.68	44.14	15
			Unilateral (right side)	438.06	60.28	15
	200 N	Not splinted	Bilateral	1366.61	40.27	15

	(single-units)	Anterior	1438.87	49.26	15
		Unilateral (right side)	1459.96	47.85	15
	Splinted	Bilateral	1224.28	39.40	15
		Anterior	1313.13	41.03	15
		Unilateral (right side)	1293.81	29.82	15
250 N	Not splinted (single-units)	Bilateral	1455.42	68.06	15
		Anterior	1543.51	45.16	15
		Unilateral (right side)	1527.20	47.14	15
	Splinted	Bilateral	1288.16	30.13	15
		Anterior	1615.88	52.24	15
		Unilateral (right side)	1390.99	32.18	15
300 N	Not splinted (single-units)	Bilateral	1665.11	47.52	15
		Unilateral (right side)	1632.34	99.17	15
	Splinted	Bilateral	1462.59	40.47	15
		Unilateral (right side)	1485.95	62.58	15

N= number

Supplementary Table 10. Multivariate analysis of periimplant microstrains in the four-MDI models dependent on the loading position (bilateral, anterior and unilateral-right-side), splinting status (single-units or splinted MDIs), as well as on the Loading force (50-300 N)

Multivariate analysis - 4 MDIs: Tests of Between-Subjects Effects

Source	**Strain Gauge Position**	**Type III Sum of Squares**	**df**	**Mean Square**	**F**	***P***	**Partial Eta Squared**
Corrected Model	Right Posterior MDI Vestibular SG	154120405.857[a]	33	4670315.33	1239.45	<0.001	0.99
	Left Posterior MDI Vestibular SG	153404665.214[b]	33	4648626.22	2321.59	<0.001	0.99
	Right Posterior MDI Oral SG	153325663.823[c]	33	4646232.24	1517.45	<0.001	0.99
	Left Posterior MDI Oral SG	119971349.049[d]	33	3635495.43	2116.56	<0.001	0.99
	Right Anterior MDI Vestibular SG	147944978.160[e]	33	4483181.16	1739.17	<0.001	0.99
	Left Anterior MDI Vestibular SG	147757994.667[f]	33	4477514.99	1828.82	<0.001	0.99
	Right Anterior MDI Oral SG	147122661.453[g]	33	4458262.47	1573.39	<0.001	0.99
	Left Anterior MDI Oral SG	144752783.875[h]	33	4386448.00	1928.98	<0.001	0.99
Intercept	Right Posterior MDI Vestibular SG	677172012.98	1	677172012.98	179713.40	<0.001	1.00
	Left Posterior MDI Vestibular SG	534136859.76	1	534136859.76	266755.60	<0.001	1.00
	Right Posterior MDI Oral SG	580668748.58	1	580668748.58	189644.93	<0.001	1.00
	Left Posterior MDI Oral SG	388378938.25	1	388378938.25	226111.29	<0.001	1.00
	Right Anterior MDI Vestibular SG	594138776. 46	1	594138776.46	230484.90	<0.001	1.00
	Left Anterior MDI Vestibular SG	540295029.18	1	540295029.18	220680.83	<0.001	1.00
	Right Anterior MDI Oral SG	505661981.85	1	505661981.85	178455.55	<0.001	1.00
	Left Anterior MDI Oral SG	479743072.34	1	479743072.34	210971.77	<0.001	1.00
Force	Right Posterior MDI Vestibular SG	138870722.81	5	27774144.56	7370.93	<0.001	0.99
	Left Posterior MDI Vestibular SG	135791489.20	5	27158297.84	13563.24	<0.001	0.99
	Right Posterior MDI Oral SG	135672211.99	5	27134442.40	8862.04	<0.001	0.99
	Left Posterior MDI Oral SG	95154202.81	5	19030840.56	11079.61	<0.001	0.99
	Right Anterior MDI Vestibular SG	133723045.84	5	26744609.17	10375.07	<0.001	0.99
	Left Anterior MDI Vestibular SG	134295957.74	5	26859191.55	10970.50	<0.001	0.99
	Right Anterior MDI Oral SG	132021652.33	5	26404330.47	9318.48	<0.001	0.99
	Left Anterior MDI Oral SG	131541073.98	5	26308214.80	11569.30	<0.001	0.99
Splinting status	Right Posterior MDI Vestibular SG	1809166.50	1	1809166.50	480.13	<0.001	0.50
	Left Posterior MDI Vestibular SG	1836274.31	1	1836274.31	917.06	<0.001	0.66
	Right Posterior MDI Oral SG	1579078.73	1	1579078.73	515.72	<0.001	0.52
	Left Posterior MDI Oral SG	11836227.32	1	11836227.32	6890.96	<0.001	0.94
	Right Anterior MDI Vestibular SG	1629959.66	1	1629959.66	632.31	<0.001	0.57
	Left Anterior MDI Vestibular SG	993009.22	1	993009.22	405.59	<0.001	0.46

	Right Anterior MDI Oral SG	2203831.06	1	2203831.06	777.76	<0.001	0.62
	Left Anterior MDI Oral SG	2011899.31	1	2011899.31	884.75	<0.001	0.65
Loading position	Right Posterior MDI Vestibular SG	2233275.88	2	1116637.94	296.34	<0.001	0.55
	Left Posterior MDI Vestibular SG	8705395.64	2	4352697.82	2173.80	<0.001	0.90
	Right Posterior MDI Oral SG	2688825.89	2	1344412.94	439.08	<0.001	0.65
	Left Posterior MDI Oral SG	2521354.90	2	1260677.45	733.96	<0.001	0.76
	Right Anterior MDI Vestibular SG	9288871.45	2	4644435.73	1801.72	<0.001	0.88
	Left Anterior MDI Vestibular SG	11061100.84	2	5530550.42	2258.93	<0.001	0.90
	Right Anterior MDI Oral SG	7094326.52	2	3547163.26	1251.85	<0.001	0.84
	Left Anterior MDI Oral SG	6217683.50	2	3108841.75	1367.14	<0.001	0.85
Force * Splinting status	Right Posterior MDI Vestibular SG	65757.86	5	13151.57	3.49	0.004	0.04
	Left Posterior MDI Vestibular SG	111153.33	5	22230.67	11.10	<0.001	0.10
	Right Posterior MDI Oral SG	165279.20	5	33055.84	10.80	<0.001	0.10
	Left Posterior MDI Oral SG	4157604.06	5	831520.81	484.11	<0.001	0.84
	Right Anterior MDI Vestibular SG	64655.92	5	12931.18	5.02	<0.001	0.05
	Left Anterior MDI Vestibular SG	8356.13	5	1671.23	0.68	0.637	0.01
	Right Anterior MDI Oral SG	271597.64	5	54319.53	19.17	<0.001	0.17
	Left Anterior MDI Oral SG	134659.85	5	26931.97	11.84	<0.001	0.11
Force * Loading position	Right Posterior MDI Vestibular SG	6589158.88	9	732128.76	194.30	<0.001	0.79
	Left Posterior MDI Vestibular SG	8404541.76	9	933837.97	466.37	<0.001	0.90
	Right Posterior MDI Oral SG	8209678.28	9	912186.48	297.92	<0.001	0.85
	Left Posterior MDI Oral SG	7024122.37	9	780458.04	454.38	<0.001	0.90
	Right Anterior MDI Vestibular SG	7968244.52	9	885360.50	343.46	<0.001	0.87
	Left Anterior MDI Vestibular SG	7840502.11	9	871166.90	355.82	<0.001	0.87
	Right Anterior MDI Oral SG	8199158.61	9	911017.62	321.51	<0.001	0.86
	Left Anterior MDI Oral SG	8657901.66	9	961989.07	423.04	<0.001	0.89
Splinting status * Loading Position	Right Posterior MDI Vestibular SG	27627.38	2	13813.69	3.67	0.026	0.02
	Left Posterior MDI Vestibular SG	2805.39	2	1402.70	0.70	0.497	0.00
	Right Posterior MDI Oral SG	57097.37	2	28548.68	9.32	<0.001	0.04
	Left Posterior MDI Oral SG	120434.87	2	60217.44	35.06	<0.001	0.13
	Right Anterior MDI Vestibular SG	213934.64	2	106967.32	41.50	<0.001	0.15
	Left Anterior MDI Vestibular SG	207161.13	2	103580.57	42.31	<0.001	0.15
	Right Anterior MDI Oral SG	285627.30	2	142813.65	50.40	<0.001	0.17
	Left Anterior MDI Oral SG	325835.44	2	162917.72	71.64	<0.001	0.23
Force * Splinting status * Loading position	Right Posterior MDI Vestibular SG	198301.95	9	22033.55	5.85	<0.001	0.10
	Left Posterior MDI Vestibular SG	581933.57	9	64659.29	32.29	<0.001	0.38
	Right Posterior MDI Oral SG	226491.76	9	25165.75	8.22	<0.001	0.13
	Left Posterior MDI Oral SG	446488.97	9	49609.89	28.88	<0.001	0.35

	Right Anterior MDI Vestibular SG	102666.55	9	11407.39	4.43	<0.001	0.08
	Left Anterior MDI Vestibular SG	229558.04	9	25506.45	10.42	<0.001	0.16
	Right Anterior MDI Oral SG	227745.02	9	25305.00	8.93	<0.001	0.14
	Left Anterior MDI Oral SG	234851.52	9	26094.61	11.48	<0.001	0.18
Error	Right Posterior MDI Vestibular SG	1793599.61	476	3768.07			
	Left Posterior MDI Vestibular SG	953116.44	476	2002.35			
	Right Posterior MDI Oral SG	1457451.66	476	3061.87			
	Left Posterior MDI Oral SG	817599.04	476	1717.65			
	Right Anterior MDI Vestibular SG	1227022.05	476	2577.78			
	Left Anterior MDI Vestibular SG	1165395.42	476	2448.31			
	Right Anterior MDI Oral SG	1348767.85	476	2833.55			
	Left Anterior MDI Oral SG	1082408.82	476	2273.97			
Total	Right Posterior MDI Vestibular SG	816322653.76	510				
	Left Posterior MDI Vestibular SG	666629327.44	510				
	Right Posterior MDI Oral SG	721408276.10	510				
	Left Posterior MDI Oral SG	496458648.54	510				
	Right Anterior MDI Vestibular SG	717726278.08	510				
	Left Anterior MDI Vestibular SG	661920755.90	510				
	Right Anterior MDI Oral SG	632745975.01	510				
	Left Anterior MDI Oral SG	603155346.26	510				
Corrected Total	Right Posterior MDI Vestibular SG	155914005.46	509				
	Left Posterior MDI Vestibular SG	154357781.66	509				
	Right Posterior MDI Oral SG	154783115.48	509				
	Left Posterior MDI Oral SG	120788948.09	509				
	Right Anterior MDI Vestibular SG	149172000.21	509				
	Left Anterior MDI Vestibular SG	148923390.09	509				
	Right Anterior MDI Oral SG	148471429.30	509				
	Left Anterior MDI Oral SG	145835192.69	509				

a. R Squared = .988 (Adjusted R Squared = .988)
b. R Squared = .994 (Adjusted R Squared = .993)
c. R Squared = .991 (Adjusted R Squared = .990)
d. R Squared = .993 (Adjusted R Squared = .993)
e. R Squared = .992 (Adjusted R Squared = .991)
f. R Squared = .992 (Adjusted R Squared = .992)
g. R Squared = .991 (Adjusted R Squared = .990)
h. R Squared = .993 (Adjusted R Squared = .992)

df=degree of freedom, *P*=level of significance, F=F value

Supplementary Table 11. Post-hoc Sheffe: Periimplant microstrains dependent on different Loading forces in the four-MDI models

Multiple Comparisons 4 MDIs: Periimplant Microstrains

Post-hoc Scheffe

Dependent Variable	(I) Force	(J) Force	Mean Difference (I-J)	*P*	95% Confidence Interval	
					Lower Bound	Upper Bound
Right Posterior MDI Vestibular SG	50 N	100 N	-183.2643*	<0.001	-213.84	-152.69
		150 N	-594.1338*	<0.001	-624.71	-563.56
		200 N	-1049.7246*	<0.001	-1080.30	-1019.15
		250 N	-1227.1257*	<0.001	-1257.70	-1196.55
		300 N	-1497.1273*	<0.001	-1531.31	-1462.94
	100 N	50 N	183.2643*	<0.001	152.69	213.84
		150 N	-410.8695*	<0.001	-441.45	-380.29
		200 N	-866.4603*	<0.001	-897.04	-835.88
		250 N	-1043.8614*	<0.001	-1074.44	-1013.29
		300 N	-1313.8630*	<0.001	-1348.05	-1279.68
	150 N	50 N	594.1338*	<0.001	563.56	624.71
		100 N	410.8695*	<0.001	380.29	441.45
		200 N	-455.5908*	<0.001	-486.17	-425.02
		250 N	-632.9918*	<0.001	-663.57	-602.42
		300 N	-902.9934*	<0.001	-937.18	-868.81
	200 N	50 N	1049.7246*	<0.001	1019.15	1080.30
		100 N	866.4603*	<0.001	835.88	897.04
		150 N	455.5908*	<0.001	425.02	486.17
		250 N	-177.4011*	<0.001	-207.98	-146.83
		300 N	-447.4027*	<0.001	-481.59	-413.22
	250 N	50 N	1227.1257*	<0.001	1196.55	1257.70
		100 N	1043.8614*	<0.001	1013.29	1074.44
		150 N	632.9918*	<0.001	602.42	663.57
		200 N	177.4011*	<0.001	146.83	207.98
		300 N	-270.0016*	<0.001	-304.19	-235.82
	300 N	50 N	1497.1273*	<0.001	1462.94	1531.31
		100 N	1313.8630*	<0.001	1279.68	1348.05
		150 N	902.9934*	<0.001	868.81	937.18
		200 N	447.4027*	<0.001	413.22	481.59
		250 N	270.0016*	<0.001	235.82	304.19
Left Posterior MDI Vestibular SG	50 N	100 N	-167.0960*	<0.001	-189.38	-144.81
		150 N	-561.9220*	<0.001	-584.21	-539.63
		200 N	-1052.2012*	<0.001	-1074.49	-1029.91
		250 N	-1208.9913*	<0.001	-1231.28	-1186.70
		300 N	-1381.2308*	<0.001	-1406.15	-1356.31
	100 N	50 N	167.0960*	<0.001	144.81	189.38
		150 N	-394.8259*	<0.001	-417.11	-372.54
		200 N	-885.1052*	<0.001	-907.39	-862.82
		250 N	-1041.8952*	<0.001	-1064.18	-1019.61
		300 N	-1214.1348*	<0.001	-1239.05	-1189.22
	150 N	50 N	561.9220*	<0.001	539.63	584.21
		100 N	394.8259*	<0.001	372.54	417.11
		200 N	-490.2792*	<0.001	-512.57	-467.99
		250 N	-647.0693*	<0.001	-669.36	-624.78
		300 N	-819.3088*	<0.001	-844.23	-794.39

	200 N	50 N	1052.2012*	<0.001	1029.91	1074.49
		100 N	885.1052*	<0.001	862.82	907.39
		150 N	490.2792*	<0.001	467.99	512.57
		250 N	-156.7901*	<0.001	-179.08	-134.50
		300 N	-329.0296*	<0.001	-353.95	-304.11
	250 N	50 N	1208.9913*	<0.001	1186.70	1231.28
		100 N	1041.8952*	<0.001	1019.61	1064.18
		150 N	647.0693*	<0.001	624.78	669.36
		200 N	156.7901*	<0.001	134.50	179.08
		300 N	-172.2395*	<0.001	-197.16	-147.32
	300 N	50 N	1381.2308*	<0.001	1356.31	1406.15
		100 N	1214.1348*	<0.001	1189.22	1239.05
		150 N	819.3088*	<0.001	794.39	844.23
		200 N	329.0296*	<0.001	304.11	353.95
		250 N	172.2395*	<0.001	147.32	197.16
**Right Posterior MDI Oral SG**	50 N	100 N	-164.7145*	<0.001	-192.28	-137.15
		150 N	-563.6795*	<0.001	-591.24	-536.12
		200 N	-1047.8388*	<0.001	-1075.40	-1020.28
		250 N	-1216.8798*	<0.001	-1244.44	-1189.32
		300 N	-1452.6451*	<0.001	-1483.46	-1421.83
	100 N	50 N	164.7145*	<0.001	137.15	192.28
		150 N	-398.9650*	<0.001	-426.53	-371.40
		200 N	-883.1243*	<0.001	-910.69	-855.56
		250 N	-1052.1653*	<0.001	-1079.73	-1024.60
		300 N	-1287.9306*	<0.001	-1318.75	-1257.12
	150 N	50 N	563.6795*	<0.001	536.12	591.24
		100 N	398.9650*	<0.001	371.40	426.53
		200 N	-484.1592*	<0.001	-511.72	-456.60
		250 N	-653.2003*	<0.001	-680.76	-625.64
		300 N	-888.9656*	<0.001	-919.78	-858.15
	200 N	50 N	1047.8388*	<0.001	1020.28	1075.40
		100 N	883.1243*	<0.001	855.56	910.69
		150 N	484.1592*	<0.001	456.60	511.72
		250 N	-169.0410*	<0.001	-196.60	-141.48
		300 N	-404.8064*	<0.001	-435.62	-373.99
	250 N	50 N	1216.8798*	<0.001	1189.32	1244.44
		100 N	1052.1653*	<0.001	1024.60	1079.73
		150 N	653.2003*	<0.001	625.64	680.76
		200 N	169.0410*	<0.001	141.48	196.60
		300 N	-235.7654*	<0.001	-266.58	-204.95
	300 N	50 N	1452.6451*	<0.001	1421.83	1483.46
		100 N	1287.9306*	<0.001	1257.12	1318.75
		150 N	888.9656*	<0.001	858.15	919.78
		200 N	404.8064*	<0.001	373.99	435.62
		250 N	235.7654*	<0.001	204.95	266.58
**Left Posterior MDI Oral SG**	50 N	100 N	-148.1632*	<0.001	-168.81	-127.52
		150 N	-491.4191*	<0.001	-512.06	-470.78
		200 N	-951.9745*	<0.001	-972.62	-931.33
		250 N	-1012.7456*	<0.001	-1033.39	-992.10
		300 N	-1117.9993*	<0.001	-1141.08	-1094.92
	100 N	50 N	148.1632*	<0.001	127.52	168.81
		150 N	-343.2559*	<0.001	-363.90	-322.61
		200 N	-803.8113*	<0.001	-824.45	-783.17
		250 N	-864.5825*	<0.001	-885.23	-843.94
		300 N	-969.8361*	<0.001	-992.92	-946.76
	150 N	50 N	491.4191*	<0.001	470.78	512.06

		100 N	343.2559*	<0.001	322.61	363.90
		200 N	-460.5554*	<0.001	-481.20	-439.91
		250 N	-521.3265*	<0.001	-541.97	-500.68
		300 N	-626.5802*	<0.001	-649.66	-603.50
	200 N	50 N	951.9745*	<0.001	931.33	972.62
		100 N	803.8113*	<0.001	783.17	824.45
		150 N	460.5554*	<0.001	439.91	481.20
		250 N	-60.7711*	<0.001	-81.41	-40.13
		300 N	-166.0248*	<0.001	-189.11	-142.94
	250 N	50 N	1012.7456*	<0.001	992.10	1033.39
		100 N	864.5825*	<0.001	843.94	885.23
		150 N	521.3265*	<0.001	500.68	541.97
		200 N	60.7711*	<0.001	40.13	81.41
		300 N	-105.2537*	<0.001	-128.33	-82.17
	300 N	50 N	1117.9993*	<0.001	1094.92	1141.08
		100 N	969.8361*	<0.001	946.76	992.92
		150 N	626.5802*	<0.001	603.50	649.66
		200 N	166.0248*	<0.001	142.94	189.11
		250 N	105.2537*	<0.001	82.17	128.33
**Right Anterior MDI Vestibular SG**	50 N	100 N	-138.2003*	<0.001	-163.49	-112.91
		150 N	-534.8862*	<0.001	-560.18	-509.60
		200 N	-1046.1013*	<0.001	-1071.39	-1020.81
		250 N	-1188.9117*	<0.001	-1214.20	-1163.62
		300 N	-1304.4991*	<0.001	-1332.77	-1276.22
	100 N	50 N	138.2003*	<0.001	112.91	163.49
		150 N	-396.6859*	<0.001	-421.98	-371.40
		200 N	-907.9011*	<0.001	-933.19	-882.61
		250 N	-1050.7114*	<0.001	-1076.00	-1025.42
		300 N	-1166.2989*	<0.001	-1194.57	-1138.02
	150 N	50 N	534.8862*	<0.001	509.60	560.18
		100 N	396.6859*	<0.001	371.40	421.98
		200 N	-511.2152*	<0.001	-536.50	-485.93
		250 N	-654.0255*	<0.001	-679.32	-628.74
		300 N	-769.6130*	<0.001	-797.89	-741.34
	200 N	50 N	1046.1013*	<0.001	1020.81	1071.39
		100 N	907.9011*	<0.001	882.61	933.19
		150 N	511.2152*	<0.001	485.93	536.50
		250 N	-142.8104*	<0.001	-168.10	-117.52
		300 N	-258.3978*	<0.001	-286.67	-230.12
	250 N	50 N	1188.9117*	<0.001	1163.62	1214.20
		100 N	1050.7114*	<0.001	1025.42	1076.00
		150 N	654.0255*	<0.001	628.74	679.32
		200 N	142.8104*	<0.001	117.52	168.10
		300 N	-115.5874*	<0.001	-143.86	-87.31
	300 N	50 N	1304.4991*	<0.001	1276.22	1332.77
		100 N	1166.2989*	<0.001	1138.02	1194.57
		150 N	769.6130*	<0.001	741.34	797.89
		200 N	258.3978*	<0.001	230.12	286.67
		250 N	115.5874*	<0.001	87.31	143.86
**Left Anterior MDI Vestibular SG**	50 N	100 N	-117.4073*	<0.001	-142.05	-92.76
		150 N	-521.6674*	<0.001	-546.31	-497.02
		200 N	-1049.2590*	<0.001	-1073.91	-1024.61
		250 N	-1170.8817*	<0.001	-1195.53	-1146.24
		300 N	-1276.5839*	<0.001	-1304.14	-1249.03

	100 N	50 N	117.4073*	<0.001	92.76	142.05
		150 N	-404.2601*	<0.001	-428.91	-379.61
		200 N	-931.8517*	<0.001	-956.50	-907.21
		250 N	-1053.4744*	<0.001	-1078.12	-1028.83
		300 N	-1159.1766*	<0.001	-1186.73	-1131.62
	150 N	50 N	521.6674*	<0.001	497.02	546.31
		100 N	404.2601*	<0.001	379.61	428.91
		200 N	-527.5916*	<0.001	-552.24	-502.95
		250 N	-649.2143*	<0.001	-673.86	-624.57
		300 N	-754.9165*	<0.001	-782.47	-727.36
	200 N	50 N	1049.2590*	<0.001	1024.61	1073.91
		100 N	931.8517*	<0.001	907.21	956.50
		150 N	527.5916*	<0.001	502.95	552.24
		250 N	-121.6227*	<0.001	-146.27	-96.98
		300 N	-227.3249*	<0.001	-254.88	-199.77
	250 N	50 N	1170.8817*	<0.001	1146.24	1195.53
		100 N	1053.4744*	<0.001	1028.83	1078.12
		150 N	649.2143*	<0.001	624.57	673.86
		200 N	121.6227*	<0.001	96.98	146.27
		300 N	-105.7022*	<0.001	-133.26	-78.15
	300 N	50 N	1276.5839*	<0.001	1249.03	1304.14
		100 N	1159.1766*	<0.001	1131.62	1186.73
		150 N	754.9165*	<0.001	727.36	782.47
		200 N	227.3249*	<0.001	199.77	254.88
		250 N	105.7022*	<0.001	78.15	133.26
**Right Anterior MDI Oral SG**	50 N	100 N	-143.5333*	<0.001	-170.05	-117.02
		150 N	-489.2701*	<0.001	-515.78	-462.76
		200 N	-1045.6853*	<0.001	-1072.20	-1019.17
		250 N	-1193.5697*	<0.001	-1220.08	-1167.06
		300 N	-1290.5056*	<0.001	-1320.15	-1260.86
	100 N	50 N	143.5333*	<0.001	117.02	170.05
		150 N	-345.7368*	<0.001	-372.25	-319.22
		200 N	-902.1521*	<0.001	-928.67	-875.64
		250 N	-1050.0364*	<0.001	-1076.55	-1023.52
		300 N	-1146.9723*	<0.001	-1176.62	-1117.33
	150 N	50 N	489.2701*	<0.001	462.76	515.78
		100 N	345.7368*	<0.001	319.22	372.25
		200 N	-556.4152*	<0.001	-582.93	-529.90
		250 N	-704.2995*	<0.001	-730.81	-677.79
		300 N	-801.2354*	<0.001	-830.88	-771.59
	200 N	50 N	1045.6853*	<0.001	1019.17	1072.20
		100 N	902.1521*	<0.001	875.64	928.67
		150 N	556.4152*	<0.001	529.90	582.93
		250 N	-147.8843*	<0.001	-174.40	-121.37
		300 N	-244.8202*	<0.001	-274.46	-215.18
	250 N	50 N	1193.5697*	<0.001	1167.06	1220.08
		100 N	1050.0364*	<0.001	1023.52	1076.55
		150 N	704.2995*	<0.001	677.79	730.81
		200 N	147.8843*	<0.001	121.37	174.40
		300 N	-96.9359*	<0.001	-126.58	-67.29
	300 N	50 N	1290.5056*	<0.001	1260.86	1320.15
		100 N	1146.9723*	<0.001	1117.33	1176.62
		150 N	801.2354*	<0.001	771.59	830.88
		200 N	244.8202*	<0.001	215.18	274.46

		250 N	96.9359*	<0.001	67.29	126.58
Left Anterior MDI Oral SG	50 N	100 N	-129.0930*	<0.001	-152.85	-105.34
		150 N	-490.1093*	<0.001	-513.86	-466.36
		200 N	-1054.0463*	<0.001	-1077.80	-1030.29
		250 N	-1174.7974*	<0.001	-1198.55	-1151.04
		300 N	-1266.1025*	<0.001	-1292.66	-1239.55
	100 N	50 N	129.0930*	<0.001	105.34	152.85
		150 N	-361.0163*	<0.001	-384.77	-337.26
		200 N	-924.9533*	<0.001	-948.71	-901.20
		250 N	-1045.7044*	<0.001	-1069.46	-1021.95
		300 N	-1137.0095*	<0.001	-1163.57	-1110.45
	150 N	50 N	490.1093*	<0.001	466.36	513.86
		100 N	361.0163*	<0.001	337.26	384.77
		200 N	-563.9370*	<0.001	-587.69	-540.18
		250 N	-684.6881*	<0.001	-708.44	-660.94
		300 N	-775.9932*	<0.001	-802.55	-749.44
	200 N	50 N	1054.0463*	<0.001	1030.29	1077.80
		100 N	924.9533*	<0.001	901.20	948.71
		150 N	563.9370*	<0.001	540.18	587.69
		250 N	-120.7511*	<0.001	-144.50	-97.00
		300 N	-212.0562*	<0.001	-238.61	-185.50
	250 N	50 N	1174.7974*	<0.001	1151.04	1198.55
		100 N	1045.7044*	<0.001	1021.95	1069.46
		150 N	684.6881*	<0.001	660.94	708.44
		200 N	120.7511*	<0.001	97.00	144.50
		300 N	-91.3051*	<0.001	-117.86	-64.75
	300 N	50 N	1266.1025*	<0.001	1239.55	1292.66
		100 N	1137.0095*	<0.001	1110.45	1163.57
		150 N	775.9932*	<0.001	749.44	802.55
		200 N	212.0562*	<0.001	185.50	238.61
		250 N	91.3051*	<0.001	64.75	117.86

Based on observed means.
The error term is Mean Square(Error) = 2273.968.
*. The mean difference is significant at the .05 level.

Supplementary Table 12. Multiple comparisons: post-hoc tests (Sheffe): Significance of the differences of periimplant microstrains in the four-MDI models during the mandibular overdenture loading at different loading positions (Bilaterally, anteriorly and unilaterally-right side)

Multiple Comparisons- 4 MDIs: Posthoc Sheffe						
Strain Gauge Position	**Loading Position**		**Mean Difference (I-J)**	***P***	**95% Confidence Interval**	
					**Lower Bound**	**Upper Bound**
**Right Posterior MDI Vestibular SG**	Bilateral	Anterior	141.7680*	<0.001	125.10	158.43
		Unilateral (right side)	-140.2863*	<0.001	-156.17	-124.40
	Anterior	Bilateral	-141.7680*	<0.001	-158.43	-125.10
		Unilateral (right side)	-282.0543*	<0.001	-298.72	-265.39
	Unilateral (right side)	Bilateral	140.2863*	<0.001	124.40	156.17
		Anterior	282.0543*	<0.001	265.39	298.72
**Left Posterior MDI Vestibular SG**	Bilateral	Anterior	127.1023*	<0.001	114.95	139.25
		Unilateral (right side)	269.9385*	<0.001	258.36	281.52
	Anterior	Bilateral	-127.1023*	<0.001	-139.25	-114.95
		Unilateral (right side)	142.8363*	<0.001	130.69	154.98
	Unilateral (right side)	Bilateral	-269.9385*	<0.001	-281.52	-258.36
		Anterior	-142.8363*	<0.001	-154.98	-130.69
**Right Posterior MDI Oral SG**	Bilateral	Anterior	162.1360*	<0.001	147.11	177.16
		Unilateral (right side)	-140.5882*	<0.001	-154.91	-126.27
	Anterior	Bilateral	-162.1360*	<0.001	-177.16	-147.11
		Unilateral (right side)	-302.7241*	<0.001	-317.75	-287.70
	Unilateral (right side)	Bilateral	140.5882*	<0.001	126.27	154.91
		Anterior	302.7241*	<0.001	287.70	317.75
**Left Posterior MDI Oral SG**	Bilateral	Anterior	84.0825*	<0.001	72.83	95.33
		Unilateral (right side)	138.2336*	<0.001	127.51	148.96
	Anterior	Bilateral	-84.0825*	<0.001	-95.33	-72.83
		Unilateral (right side)	54.1511*	<0.001	42.90	65.40
	Unilateral (right side)	Bilateral	-138.2336*	<0.001	-148.96	-127.51
		Anterior	-54.1511*	<0.001	-65.40	-42.90
**Right Anterior MDI Vestibular SG**	Bilateral	Anterior	-208.9124*	<0.001	-222.69	-195.13
		Unilateral (right side)	-167.1245*	<0.001	-180.27	-153.98
	Anterior	Bilateral	208.9124*	<0.001	195.13	222.69
		Unilateral (right side)	41.7878*	<0.001	28.01	55.57
	Unilateral (right side)	Bilateral	167.1245*	<0.001	153.98	180.27
		Anterior	-41.7878*	<0.001	-55.57	-28.01
**Left Anterior**	Bilateral	Anterior	-206.8005*	<0.001	-220.23	-193.37
		Unilateral (right side)	-21.4844*	<0.001	-34.29	-8.68
	Anterior	Bilateral	206.8005*	<0.001	193.37	220.23

**MDI Vestibular SG**		Unilateral (right side)	185.3161*	<0.001	171.88	198.75
	Unilateral (right side)	Bilateral	21.4844*	<0.001	8.68	34.29
		Anterior	-185.3161*	<0.001	-198.75	-171.88
**Right Anterior MDI Oral SG**	Bilateral	Anterior	-159.9785*	<0.001	-174.43	-145.53
		Unilateral (right side)	-192.6138*	<0.001	-206.39	-178.84
	Anterior	Bilateral	159.9785*	<0.001	145.53	174.43
		Unilateral (right side)	-32.6353*	<0.001	-47.09	-18.19
	Unilateral (right side)	Bilateral	192.6138*	<0.001	178.84	206.39
		Anterior	32.6353*	<0.001	18.19	47.09
**Left Anterior MDI Oral SG**	Bilateral	Anterior	-143.0103*	<0.001	-155.96	-130.07
		Unilateral (right side)	-59.3493*	<0.001	-71.69	-47.01
	Anterior	Bilateral	143.0103*	<0.001	130.07	155.96
		Unilateral (right side)	83.6610*	<0.001	70.72	96.61
	Unilateral (right side)	Bilateral	59.3493*	<0.001	47.01	71.69
		Anterior	-83.6610*	<0.001	-96.61	-70.72

Based on observed means.
The error term is Mean Square(Error) = 2273.968.
***. The mean difference is significant at the .05 level.**

Supplementary Table 13. Descriptive statistics: Microstrains registered from the right-side and left-side posterior edentulous areas under mandibular overdenture supported by two mini-implants (MDIs), either not-splinted (single-units) or splinted, when overdenture was loaded bilaterally and unilaterally with 50, 100, 150, 200, 250, and 300 N forces

Posterior Edentulous Area	*Loading Position*	*Force*	*Splinting status*	*Mean*	*Standard Deviation*	*N*
Right Side	**Bilateral loading**	50 N	Single-Units (not Splinted)	**394.73**	66.99	15
			Splinted	**455.75**	50.21	15
		100 N	Single-Units (not Splinted)	**616.45**	82.76	15
			Splinted	**648.02**	50.03	15
		150 N	(not Splinted)	**1054.94**	113.07	15
			Splinted	**925.19**	54.20	15
		200 N	Single-Units (not Splinted)	**939.91**	66.77	15
			Splinted	**1004.76**	52.98	15
		250 N	Single-Units (not Splinted)	**1172.30**	92.32	15
			Splinted	**1203.03**	64.20	15
		300 N	Single-Units (not Splinted)	**1602.41**	114.58	15
			Splinted	**1471.84**	69.72	15
	**Unilateral loading (Right Side)**	50 N	Single-Units (not Splinted)	**920.93**	135.02	15
			Splinted	**709.44**	23.57	15
		100 N	Single-Units (not Splinted)	**1204.53**	128.14	15
			Splinted	**902.59**	18.18	15
		150 N	Single-Units (not Splinted)	**1360.17**	69.00	15
			Splinted	**1068.44**	59.33	15
		200 N	Single-Units (not Splinted)	**1465.52**	126.33	15
			Splinted	**1264.80**	43.41	15
		250 N	Single-Units (not Splinted)	**1734.72**	130.50	15
			Splinted	**1452.93**	35.21	15
		300 N	Single-Units (not Splinted)	**1913.86**	72.15	15
			Splinted	**1622.51**	60.79	15
Left Side	**Bilateral loading**	50 N	Single-Units (not Splinted)	**500.43**	504.68	15
			Splinted	**425.65**	55.93	15
		100 N	Single-Units (not Splinted)	**637.07**	60.60	15
			Splinted	**645.25**	79.87	15
		150 N	Single-Units (not Splinted)	**1083.69**	136.50	15
			Splinted	**959.18**	114.64	15
		200 N	Single-Units (not Splinted)	**1037.07**	519.05	15
			Splinted	**971.69**	53.00	15
		250 N	Single-Units (not Splinted)	**1189.61**	63.17	15
			Splinted	**1178.84**	98.95	15
		300 N	Single-Units (not Splinted)	**1625.63**	148.33	15
			Splinted	**1499.83**	115.91	15
	**Unilateral loading (Right Side)**	50 N	Single-Units (not Splinted)	**582.31**	97.80	15
			Splinted	**134.89**	12.57	15
		100 N	Single-Units (not Splinted)	**655.16**	93.15	15
			Splinted	**274.63**	48.74	15
		150 N	Single-Units (not Splinted)	**756.90**	76.98	15
			Splinted	**413.79**	19.97	15
		200 N	Single-Units (not Splinted)	**1130.73**	98.43	15
			Splinted	**671.73**	37.10	15
		250 N	Single-Units (not Splinted)	**1193.55**	98.31	15

		Splinted	**828.40**	54.69	15
	300 N	Single-Units (not Splinted)	**1310.00**	75.32	15
		Splinted	**956.25**	30.88	15

Supplementary Table 14. Microstrains registered from the right-side and left-side posterior edentulous area under mandibular overdenture supported by three mini-implants (MDIs), either not-splinted (single-units) or splinted, when overdenture was loaded bilaterally and unilaterally with 50, 100, 150, 200, 250, and 300 N forces

Posterior Edentulous Area	*Loading Position*	*Force*	*Splinting status*	*Mean*	*Standard Deviation*	*N*
Right Side	**Bilateral loading**	50 N	Single-Units (not Splinted)	**424.72**	61.74	15
			Splinted	**400.73**	21.20	15
		100 N	Single-Units (not Splinted)	**626.65**	76.84	15
			Splinted	**610.56**	24.36	15
		150 N	Single-Units (not Splinted)	**842.94**	71.21	15
			Splinted	**859.47**	11.25	15
		200 N	Single-Units (not Splinted)	**964.99**	58.60	15
			Splinted	**947.87**	44.16	15
		250 N	Single-Units (not Splinted)	**1165.54**	85.17	15
			Splinted	**1156.34**	40.32	15
		300 N	Single-Units (not Splinted)	**1386.50**	82.53	15
			Splinted	**1407.15**	37.13	15
	**Unilateral loading (Right Side)**	50 N	Single-Units (not Splinted)	**834.64**	85.83	15
			Splinted	**740.34**	42.63	15
		100 N	Single-Units (not Splinted)	**978.33**	64.34	15
			Splinted	**862.44**	54.30	15
		150 N	Single-Units (not Splinted)	**1081.71**	117.38	15
			Splinted	**972.05**	58.91	15
		200 N	Single-Units (not Splinted)	**1390.33**	85.21	15
			Splinted	**1288.41**	59.32	15
		250 N	Single-Units (not Splinted)	**1521.76**	75.62	15
			Splinted	**1405.63**	49.59	15
		300 N	Single-Units (not Splinted)	**1632.90**	122.18	15
			Splinted	**1513.83**	62.84	15
Left Side	**Bilateral loading**	50 N	Single-Units (not Splinted)	**438.49**	71.75	15
			Splinted	**395.59**	28.63	15
		100 N	Single-Units (not Splinted)	**652.64**	68.96	15
			Splinted	**625.76**	47.90	15
		150 N	Single-Units (not Splinted)	**855.48**	75.62	15
			Splinted	**815.00**	41.28	15
		200 N	Single-Units (not Splinted)	**989.30**	78.56	15
			Splinted	**945.41**	28.66	15
		250 N	Single-Units (not Splinted)	**1210.18**	79.47	15
			Splinted	**1170.72**	73.05	15
		300 N	Single-Units (not Splinted)	**1415.01**	77.40	15
			Splinted	**1363.90**	38.99	15
	**Unilateral loading (Right Side)**	50 N	Single-Units (not Splinted)	**448.74**	89.82	15
			Splinted	**190.39**	46.83	15
		100 N	Single-Units (not Splinted)	**555.94**	81.70	15
			Splinted	**275.09**	59.95	15
		150 N	Single-Units (not Splinted)	**535.24**	80.13	15
			Splinted	**276.68**	45.22	15
		200 N	Single-Units (not Splinted)	**993.62**	72.43	15
			Splinted	**738.28**	53.64	15
		250 N	Single-Units (not Splinted)	**1088.78**	77.74	15
			Splinted	**824.82**	73.73	15
		300 N	Single-Units (not Splinted)	**1075.54**	83.88	15

		Splinted	**805.46**	44.89	15

Supplementary Table 15. Microstrains registered from posterior edentulous area under mandibular overdenture supported by four mini-implants (MDIs), either not-splinted (single-units) or splinted, when overdenture was loaded bilaterally and unilaterally with 50, 100, 150, 200, 250, and 300 N forces

Posterior Edentulous Area	*Loading Position*	*Force*	*Splinting status*	*Mean*	*Standard Deviation*	*N*
Right Side	**Bilateral loading**	50 N	Single-Units (not Splinted)	**400.86**	55.53	15
			Splinted	**335.17**	47.29	15
		100 N	Single-Units (not Splinted)	**835.99**	55.33	15
			Splinted	**679.61**	59.93	15
		150 N	Single-Units (not Splinted)	**924.15**	70.67	15
			Splinted	**842.37**	49.80	15
		200 N	Single-Units (not Splinted)	**941.06**	72.52	15
			Splinted	**890.91**	54.60	15
		250 N	Single-Units (not Splinted)	**1386.99**	61.93	15
			Splinted	**1219.67**	57.05	15
		300 N	Single-Units (not Splinted)	**1466.23**	73.63	15
			Splinted	**1391.01**	53.45	15
	**Unilateral loading (Right Side)**	50 N	Single-Units (not Splinted)	**739.48**	70.94	15
			Splinted	**512.75**	24.19	15
		100 N	Single-Units (not Splinted)	**893.92**	68.64	15
			Splinted	**783.97**	27.97	15
		150 N	Single-Units (not Splinted)	**1019.08**	59.43	15
			Splinted	**937.79**	21.63	15
		200 N	Single-Units (not Splinted)	**1283.32**	82.35	15
			Splinted	**1070.35**	40.46	15
		250 N	Single-Units (not Splinted)	**1443.30**	76.83	15
			Splinted	**1330.55**	42.10	15
		300 N	Single-Units (not Splinted)	**1574.14**	80.34	15
			Splinted	**1489.43**	42.14	15
Left Side	**Bilateral loading**	50 N	Single-Units (not Splinted)	**570.17**	183.23	15
			Splinted	**423.96**	97.56	15
		100 N	Single-Units (not Splinted)	**864.96**	67.97	15
			Splinted	**731.79**	70.20	15
		150 N	Single-Units (not Splinted)	**971.61**	80.29	15
			Splinted	**890.08**	61.46	15
		200 N	Single-Units (not Splinted)	**1112.19**	190.02	15
			Splinted	**980.63**	102.74	15
		250 N	Single-Units (not Splinted)	**1415.14**	74.31	15
			Splinted	**1275.11**	74.88	15
		300 N	Single-Units (not Splinted)	**1520.18**	93.51	15
			Splinted	**1440.22**	68.86	15
	**Unilateral loading (Right Side)**	50 N	Single-Units (not Splinted)	**494.38**	74.63	15
			Splinted	**352.81**	26.91	15
		100 N	Single-Units (not Splinted)	**811.99**	59.81	15
			Splinted	**647.64**	67.54	15
		150 N	Single-Units (not Splinted)	**904.81**	64.31	15
			Splinted	**822.75**	74.88	15
		200 N	Single-Units (not Splinted)	**1039.72**	70.29	15
			Splinted	**897.75**	37.95	15
		250 N	Single-Units (not Splinted)	**1379.65**	63.82	15
			Splinted	**1193.90**	86.18	15
		300 N	Single-Units (not Splinted)	**1456.78**	64.44	15

	Splinted	**1385.72**	87.33	15

Supplementary Table 16. Multivariate analysis: Effects of Splinting status (splinted and not-splinted MDIs), Loading position (Bilateral and Unilateral-Right side), Extent of Applied Force (50, 100, 150, 200, 250 and 300 N, respectively), and Number of MDIs on Posterior Area Microstrains

2 MDIs: Strains from Edentulous posterior Areas: Tests of Between-Subjects Effects

Source	Dependent Variable	Type III Sum of Squares	df	Mean Square	F	Sig.	Partial Eta Squared
Corrected Model	Posterior Edentulous Area: Right Side	143654964.872[a]	71	2023309.36	414.95	<0.001**	0.967
	Posterior Edentulous Area: Left Side	137707569.663[b]	71	1939543.23	150.57	<0.001**	0.914
Intercept	Posterior Edentulous Area: Right Side	1219782001.14	1	1219782001.14	250160.03	<0.001**	0.996
	Posterior Edentulous Area: Left Side	694473220.57	1	694473220.57	53912.42	<0.001**	0.982
Loading Position	Posterior Edentulous Area: Right Side	18112016.87	1	18112016.87	3714.52	<0.001**	0.787
	Posterior Edentulous Area: Left Side	22112758.27	1	22112758.27	1716.63	<0.001**	0.630
Force	Posterior Edentulous Area: Right Side	112575316.24	5	22515063.25	4617.52	<0.001**	0.958
	Posterior Edentulous Area: Left Side	92821840.29	5	18564368.06	1441.16	<0.001**	0.877
Splinting Status	Posterior Edentulous Area: Right Side	2948905.52	1	1269012.82	369.21	<0.001**	0.375
	Posterior Edentulous Area: Left Side	8008100.59	1	1652456.28	274.116	<0.001**	0.381
Number of mini-implants (MDIs)	Posterior Edentulous Area: Right Side	2533232.70	2	1266616.35	259.77	<0.001**	0.340
	Posterior Edentulous Area: Left Side	1909899.83	2	954949.92	74.13	<0.001**	0.128
*Loading Position * Force*	Posterior Edentulous Area: Right Side	1395774.76	5	279154.95	57.25	<0.001**	0.221
	Posterior Edentulous Area: Left Side	5358742.06	5	1071748.41	83.20	<0.001**	0.292
*Loading Position * Splinting Status*	Posterior Edentulous Area: Right Side	1166733.73	1	1166733.73	239.28	<0.001**	0.192
	Posterior Edentulous Area: Left Side	2339144.34	1	2339144.34	181.59	<0.001**	0.153
*Loading position * Number of mini-implants (MDIs)*	Posterior Edentulous Area: Right Side	1847399.49	2	923699.75	189.44	<0.001**	0.273
	Posterior Edentulous Area: Left Side	868502.82	2	434251.41	33.71	<0.001**	0.063
*Force * Splinting Status*	Posterior Edentulous Area: Right Side	30270.40	5	6054.08	1.24	0.287 NS	0.006
	Posterior Edentulous Area: Left Side	72636.85	5	14527.37	1.13	0.344 NS	0.006
*Force * Number of mini-implants (MDIs)*	Posterior Edentulous Area: Right Side	1132433.68	10	113243.37	23.22	<0.001**	0.187
	Posterior Edentulous Area: Left Side	1259085.61	10	125908.56	9.77	<0.001**	0.088

*Splinting status * Number of mini-implants (MDIs)*	Posterior Edentulous Area: Right Side	318361.47	2	159180.74	32.65	<0.001**	0.061
	Posterior Edentulous Area: Left Side	440883.55	2	220441.77	17.11	<0.001**	0.033
*Loading Position * Force * Splinting Status*	Posterior Edentulous Area: Right Side	56920.64	5	11384.13	2.33	0.040*	0.011
	Posterior Edentulous Area: Left Side	103207.50	5	20641.50	1.60	0.157 NS	0.008
*Loading Position * Splinting Status * Number of mini-implants (MDIs)*	Posterior Edentulous Area: Right Side	429157.37	10	42915.74	8.80	<0.001**	0.080
	Posterior Edentulous Area: Left Side	562377.63	10	56237.76	4.37	<0.001**	0.042
*Loading Position * Force * Number of mini-implants (MDIs)*	Posterior Edentulous Area: Right Side	532321.99	2	266161.00	54.59	<0.001**	0.098
	Posterior Edentulous Area: Left Side	1179828.76	2	589914.38	45.80	<0.001**	0.083
*Force * Splinting Status * Number of mini-implants (MDIs)*	Posterior Edentulous Area: Right Side	335537.45	10	33553.75	6.88	<0.001**	0.064
	Posterior Edentulous Area: Left Side	299150.31	10	29915.03	2.32	0.011*	0.023
*Loading Position * Force * Splinting Status * Number of mini-implants (MDIs*	Posterior Edentulous Area: Right Side	240582.55	10	24058.25	4.93	<0.001**	0.047
	Posterior Edentulous Area: Left Side	371411.26	10	37141.13	2.88	0.001**	0.028
Error	Posterior Edentulous Area: Right Side	4915014.87	1008	4876.01			
	Posterior Edentulous Area: Left Side	12984557.97	1008	12881.51			
Total	Posterior Edentulous Area: Right Side	1368351980.88	1080				
	Posterior Edentulous Area: Left Side	845165348.20	1080				
Corrected Total	Posterior Edentulous Area: Right Side	148569979.74	1079				
	Posterior Edentulous Area: Left Side	150692127.63	1079				

a. R Squared = .967 (Adjusted R Squared = .965)
b. R Squared = .914 (Adjusted R Squared = .908)

**=p<0.01; NS=not significant; *P*=Level of significance; df=degree of freedom; SG= strain gauge

Supplementary Table 17. Post-hoc tests (Sheffe): Significance of the Differences of Microstrains Obtained from Posterior Edentulous Area Under Different Forces Applied to Mandibular Overdentures

Posterior Edentulous Area	(I) LOADING FORCE	(J) LOADING FORCE	MEAN DIFFERENCE (I-J)	LEVEL OF SIGNIFICANCE	95% CONFIDENCE INTERVAL	
					Lower Bound	Upper Bound
Right Side	50 N	100 N	-231.1288*	<0.001	-255.67	-206.59
		150 N	-418.2293*	<0.001	-442.77	-393.69
		200 N	-548.5591*	<0.001	-573.10	-524.02
		250 N	-776.9359*	<0.001	-801.48	-752.40
		300 N	-966.8566*	<0.001	-991.40	-942.32
	100 N	50 N	231.1288*	<0.001	206.59	255.67
		150 N	-187.1005*	<0.001	-211.64	-162.56
		200 N	-317.4303*	<0.001	-341.97	-292.89
		250 N	-545.8071*	<0.001	-570.35	-521.27
		300 N	-735.7278*	<0.001	-760.27	-711.19
	150 N	50 N	418.2293*	<0.001	393.69	442.77
		100 N	187.1005*	<0.001	162.56	211.64
		200 N	-130.3298*	<0.001	-154.87	-105.79
		250 N	-358.7066*	<0.001	-383.25	-334.17
		300 N	-548.6273*	<0.001	-573.17	-524.09
	200 N	50 N	548.5591*	<0.001	524.02	573.10
		100 N	317.4303*	<0.001	292.89	341.97
		150 N	130.3298*	<0.001	105.79	154.87
		250 N	-228.3768*	<0.001	-252.92	-203.84
		300 N	-418.2975*	<0.001	-442.84	-393.76
	250 N	50 N	776.9359*	<0.001	752.40	801.48
		100 N	545.8071*	<0.001	521.27	570.35
		150 N	358.7066*	<0.001	334.17	383.25
		200 N	228.3768*	<0.001	203.84	252.92
		300 N	-189.9207*	<0.001	-214.46	-165.38
	300 N	50 N	966.8566*	<0.001	942.32	991.40
		100 N	735.7278*	<0.001	711.19	760.27
		150 N	548.6273*	<0.001	524.09	573.17
		200 N	418.2975*	<0.001	393.76	442.84
		250 N	189.9207*	<0.001	165.38	214.46
Left Side	50 N	100 N	-156.1702*	<0.001	-196.06	-116.28
		150 N	-303.5497*	<0.001	-343.44	-263.66
		200 N	-530.0708*	<0.001	-569.96	-490.19
		250 N	-688.9125*	<0.001	-728.80	-649.03
		300 N	-834.3532*	<0.001	-874.24	-794.47
	100 N	50 N	156.1702*	<0.001	116.28	196.06
		150 N	-147.3794*	<0.001	-187.26	-107.49
		200 N	-373.9006*	<0.001	-413.79	-334.02

		250 N	-532.7423*	<0.001	-572.63	-492.86
		300 N	-678.1830*	<0.001	-718.07	-638.30
	150 N	50 N	303.5497*	<0.001	263.66	343.44
		100 N	147.3794*	<0.001	107.49	187.26
		200 N	-226.5212*	<0.001	-266.41	-186.64
		250 N	-385.3628*	<0.001	-425.25	-345.48
		300 N	-530.8035*	<0.001	-570.69	-490.92
	200 N	50 N	530.0708*	<0.001	490.19	569.96
		100 N	373.9006*	<0.001	334.02	413.79
		150 N	226.5212*	<0.001	186.64	266.41
		250 N	-158.8416*	<0.001	-198.73	-118.96
		300 N	-304.2824*	<0.001	-344.17	-264.40
	250 N	50 N	688.9125*	<0.001	649.03	728.80
		100 N	532.7423*	<0.001	492.86	572.63
		150 N	385.3628*	<0.001	345.48	425.25
		200 N	158.8416*	<0.001	118.96	198.73
		300 N	-145.4407*	<0.001	-185.33	-105.56
	300 N	50 N	834.3532*	<0.001	794.47	874.24
		100 N	678.1830*	<0.001	638.30	718.07
		150 N	530.8035*	<0.001	490.92	570.69
		200 N	304.2824*	<0.001	264.40	344.17
		250 N	145.4407*	<0.001	105.56	185.33

Supplementary Table 18. Post-hoc tests (Sheffe): Significance of the Differences of Microstrains Obtained from the right-side and the left-side Posterior Edentulous Area when Different Number of Mini-Implants Supported Mandibular Overdentures

**Posterior Edentulous Area**	**Dependent Variable Number of Mini-Implants**		**Mean Difference (I-J)**	**Significance**	**95% Confidence Interval**	
					Lower Bound	Upper Bound
**Right Side**	2	3	87.2480*	<0.001	74.49	100.01
		4	113.2370*	<0.001	100.48	126.00
	3	2	-87.2480*	<0.001	-100.01	-74.49
		4	25.9890*	<0.001	13.23	38.75
	4	2	-113.2370*	<0.001	-126.00	-100.48
		3	-25.9890*	<0.001	-38.75	-13.23
**Left Side**	2	3	82.3423*	<0.001	61.60	103.08
		4	94.7690*	<0.001	74.03	115.51
	3	2	-82.3423*	<0.001	-103.08	-61.60
		4	12.43	0.34 NS	-8.31	33.16
	4	2	-94.7690*	<0.001	-115.51	-74.03
		3	-12.43	0.34 NS	-33.16	8.31
